# ﻿*Roccellinastrum*, *Cenozosia* and *Heterodermia*: Ecology and phylogeny of fog lichens and their photobionts from the coastal Atacama Desert

**DOI:** 10.3897/mycokeys.98.107764

**Published:** 2023-08-01

**Authors:** Patrick Jung, Lina Werner, Laura Briegel-Williams, Dina Emrich, Michael Lakatos

**Affiliations:** 1 University of Applied Sciences Kaiserslautern, Integrative Biotechnology, Carl-Schurz-Str. 10-16, 66953 Pirmasens, Germany University of Applied Sciences Kaiserslautern Pirmasens Germany; 2 Chair of Applied Vegetation Ecology, Faculty of Environment and Natural Resources, University of Freiburg, Tennenbacher Str. 4, 79106 Freiburg, Germany University of Freiburg Freiburg Germany

**Keywords:** Chlorolichens, *Heterodermia*, *Niebla*, Pan de Azucar, *Symbiochloris*, *Trebouxia*

## Abstract

Some deserts on Earth such as the Namib or the Atacama are influenced by fog which can lead to the formation of local fog oases - unique environments hosting a great diversity of specialized plants and lichens. Lichens of the genera *Ramalina*, *Niebla* or *Heterodermia* have taxonomically been investigated from fog oases around the globe but not from the Atacama Desert, one of the oldest and driest deserts. Conditioned by its topography and the presence of orographic fog, the National Park Pan de Azúcar in the Atacama Desert is considered to be such a lichen hotspot. Applying multi-gen loci involving phylogenetic analyses combined with intense morphological and chemical characterization, we determined the taxonomic position of five of the most abundant epiphytic lichens of this area. We evaluated *Roccellinastrumspongoideum* and *Heterodermiafollmannii* which were both described from the area but also finally showed that the genus *Cenozosia* is the endemic sister genus to *Ramalina*, *Vermilacinia*, *Namibialina* and *Niebla*. As a result, we have described the species *Heterodermiaadunca*, *C.cava* and *C.excorticata* as new lichen species. This work provides a comprehensive dataset for common fog lichen genera of the Coastal Range of the Atacama Desert that can be used as a baseline for monitoring programs and environmental health assessments.

## ﻿Introduction

Extensive coastal deserts affected by fog occur in three areas of the world, the Namib Desert of South Africa, the Sonoran Desert of Baja California and the Atacama Desert of South America - and all of them are well known to be home to a vast diversity of lichens (Nash et al. 1997; Jung et al. 2019; De los Rios et al. 2022). In these areas lichens can be of epiphytic nature, growing attached to plants (Lange et al. 2007), or saxicolous on stones (Bungartz et al. 2004) and are a frequent constituent of biological soil crusts (Lalley and Viles 2005; Jung et al. 2020a). In most cases, the occurrence of a hotspot is due to the interplay between coastal climate and topography creating so-called fog oases, where water is available for lichens as high air humidity, fog and dew instead of precipitation facilitating a unique type of vegetation made up of specialized plants and lichens (Rundel 1978; Muenchow et al. 2013). Growing under such conditions gave rise to an interesting set of lichen ecophysiological and structural adaptations such as fungal and algal stacks, large layers of epinecral fungal hyphae that act as a water reservoir, or rapid activation of photosynthetic activity following high air humidity or fog events (Lange et al. 2007; Vondrak and Kubásek 2013; Jung et al. 2019). Among the most dominant lichens of fog oases are the genera *Ramalina*, *Niebla* and *Heterodermia* (Rundel 1978; Moberg and Nash 1999; Sérusiaux et al. 2010), which are so-called chlorolichens hosting eukaryotic green algae (chlorobionts) such as members of the genus *Trebouxia*. The photobionts of such lichens have been shown to form well-separated clusters in phylogenies compared to the chlorobionts of less isolated habitats (Castillo and Beck 2012; Jung et al. 2019; Perugini et al. 2022), and the mycobionts that form the lichen thalli are also of high value for updating phylogenetic systems of large lichen complexes.

The genus *Niebla*, for example, has recently been split into *Namibialina* which is endemic to SW Africa, and a duo formed by *Niebla* and *Vermilacinia*, which are endemic to fog-influenced coastal deserts of the New World (Spjut et al. 2020). In addition, a new taxonomic outlining was created by Kondratyuk et al. (2021) for *Heterodermia*, which was then divided into *Heterodermia* s.str., *Leucodermia*, *Klauskalbia* and the polyphyletic genus *Polyblastidium*. However, this split has recently been rejected, exemplifying the complexity of this genus (de Souza et al. 2022). These studies created datasets that finally provide a sound background in order to evaluate the phylogenetic position of similar lichens from understudied coastal desert environments such as the Atacama Desert of Chile. There, orographic fog is a frequent phenomenon that allows the formation of extensive fog oases such as Las Lomitas in the National Park Pan de Azúcar, Freig Jorge or Alto Patache (Cereceda et al. 2008; Lehnert et al. 2018). Compared to the coasts of Baja California and the Namib Desert, the temperature of the Humboldt Current along the 3.500 km Coastal Range of the Atacama Desert is warmer than that of similar currents, producing high water vapor contents and consequently high aerosol liquid water content of air masses with subsequent cooling and the formation of fog called camanchaca. In addition, constant light southwest winds provide a continuous source of new aerosol water for condensation against the steep coastal ridge (Lehnert et al. 2018), which also leads to dust turbulence transporting minerals (Jung et al. 2020b), both of which promote a diverse and biomass-rich lichen flora of the coastal Atacama.

Some of the most conspicuous lichens from fog oases of the Atacama Desert are *Heterodermia*, various *Niebla* species, or *Roccellinastrum* (Follmann and Redón 1972), none of which has been included in DNA based studies. Spjut and colleagues (2020) noted in their study that *Vermilacinia* and *Niebla* species from the Atacama were described, but no DNA sequences were obtained, so that their positioning within their large phylogeny is completely uncertain. In addition, there is also the endemic *Cenozosiainanis* described from the Atacama Desert, which was once held for ‘a fistulose radiant of the South American *Nieblaceruchis*’ line by Bowler (1981). However, only a small LSU DNA sequence was created by Kistenich et al. (2018), which did not justify the phylogenetic position of the genus *Cenozosia* (Spjut et al. 2020). A similar issue exists for *Roccellinastrum*, a so-called byssoid-lichen which was taxonomically placed within the Micareaceae by Poelt (1973), but the family concept was finally disproved in 2004 (Andersen and Ekman 2004), without including DNA sequences of any *Roccellinastrum* species. This holds true till today for all lichens currently assigned to the genus such as *R.spongoideum* from the Atacama Desert, *R.epiphyllum* from Southern Chile, *R.candidum* from South America, *R.neglectum* from the needles of coniferous trees of New Zealand, and R.lagarostrobi as well as *R.flavescens* from Tasmania, all of which are deemed to be endemic (Henssen et al. 1983; Kantvilas 1996).

Not only the mycobiont’s phylogenetic position of lichens from the genera *Niebla*, *Roccellinastrum* and *Heterodermia* from the Atacama are of interest for recent advances in taxonomy but also their photobionts. During the last few years, a new phylogenetic and taxonomic framework was created for the largest chlorobiont genus *Trebouxia* based on multi-locus DNA datasets which increased the resolution and comparability of such photobiont clades (Muggia et al. 2020). However, DNA sequences from specialized fog oases lichens are largely missing since only a few studies have addressed them (Castillo and Beck 2012; Jung et al. 2019). For the lichen genus *Roccellinastrum*, for example, a ‘micareoid’ green algal photobiont has been stated without clarifying the meaning of this term or its assignment to a green algal genus (Coppins 1983). Since 1983, this description repeatedly appears for lichen photobionts (Coppins and Spribille 2004; Yahr et al. 2015; Kantvilas 2017; Launis et al. 2019), but it has only been assumed to refer to *Diplosphaerachodatii* based on the morphological description of the term (Voytsekhovich et al. 2011).

This study now aims to help fill the presented scientific gaps by an extensive evaluation of the phylogenetic position of the mycobiont and photobiont of two *Niebla* species (cf. *N.ceruchis*, cf. *N.tigrina*), two *Heterodermia* species and *Roccellinastrumspongoideum* from the National Park Pan de Azúcar, a fog oasis in the Atacama Desert. To achieve this, we critically evaluated the morphology and chemical character of the five specimens including 3D models as a reference, microscopic techniques, thin layer chromatography (TLC) and spot tests. We also created a multi-locus phylogeny following the molecular methods applied by Spjut et al. (2020) for *Niebla* and those of Kondratyuk et al. (2021) for *Heterodermia* as well as a combination of both for *Roccellinastrumspongoideum*, because no reference study existed. In addition, the partial 18S rRNA gene region of the photobionts of the lichens were sequenced and compared in a large phylogeny which allowed the assignment of trebouxioid photobiont clades including the final identification of the ‘micareoid’ photobiont.

## ﻿Materials and methods

### ﻿Sampling site

Sampling of all lichens took place in February 2022 in the National Park Pan de Azúcar in close proximity to a meteorological station (25°59'03"S, 70°36'55"W; 764 m a.s.l.). The national park is located between 25°53' and 26°15'S and 70°29' and 70°40'W along the Pacific coast in Chile, in the southern part of the Atacama Desert. A narrow pediment close to the coast characterizes the local topography with a steep ridge that reaches altitudes of up to 850 meters a.s.l. and drops towards the interior to altitudes between 700 and 400 m a.s.l. Parts of the National Park such as “Las Lomitas” are well-known as fog oases (Lehnert et al. 2018) characterized by various cacti (e.g. *Eulychniasaint-pieana*), euphorbias (*Euphorbialactiflua*) and smaller shrubs (Bernhard et al. 2018). Besides these, a diverse set of epiphytic, terricolous and saxicolous lichens, including a landscape dominated by a unique biological soil crust community termed grit crust, are a significant aspect of the vegetation and dependent on fog and dew (Lehnert et al. 2018; Jung et al. 2019; Jung et al. 2020a).The annual rainfall averages less than 13 mm, but the totals can vary, however, due to extreme rainfall events that occasionally occur in El Niño and El Niño-like years. The main water input comes from fog and dew during nights till early noon with different intensities according to seasonal shifts (Lehnert et al. 2018; Jung et al. 2020a). An average temperature of 13 °C in winter (July) and 20 °C in summer (January) with daily maxima occasionally exceeding 26 °C have been recorded as well as a relative humidity that ranges from 80% to 85% under clear skies (Rundel et al. 1996; Jung et al. 2019).

### ﻿Morphological investigations

Images of the lichens in their natural setting were obtained via photography (Panasonic Lumix 7.2), and their details were captured in the laboratory using a digital 3D 4K stereo microscope (VHX-7000, Keyence Deutschland GmbH, Neu-Isenburg, Germany) with up to 1000x magnification. Spores from apothecia were captured by homogenization of the apothecia in a drop of water on a microscope slide followed by light microscopy (BX51, Olympus, Tokyo) coupled with a camera (MicroLive, Bremen, Germany) and MicroLive 5 software (MicroLive, Bremen, Germany).

For electron microscopy, a low-temperature scanning electron microscope (Supra 55VP; Carl Zeiss, Oberkochen, Germany) was used to study fully hydrated lichen thalli. The samples were frozen in liquid nitrogen slush (K1250X Cryogenic preparation system, Quorum technologies) and mounted on special brass trays. After sublimation for 30 min at −80 °C, samples were sputter-coated with gold–palladium and viewed at a temperature of −130 °C and 5 kV accelerator voltage.

In order to create a digital, freely accessible reference for the lichens, 3D models were prepared based on the type material of each lichen used in this study by VirNat s.r.o., https://virnat.sk/, Budapest, Slovakia.

### ﻿Chemical characterization

The secondary lichen metabolites were detected from each lichen in triplicates using thin layer chromatography (TLC) following the protocol of Elix and Ernst-Russell (1993). Solvent A (toluene:dioxane:acetic acid, ratio 180:45:5), solvent B’ (hexane:methyl-tert-butyl ether:methanoic acid, ratio 140:72:18) and solvent C (toluene:acetic acid, ratio 170:30) were used. Results of TLC were analyzed by using LIAS metabolites (Elix et al. 2012).

Spot tests for each sample were applied (potassium hydroxide (K), sodium hypochlorite (C), combination of potassium hydroxide and sodium hypochlorite (KC), para-phenylendiamine (P)) and the lichen sample were also tested under UV light.

### ﻿DNA extraction

Biomass from three replicates per species were picked with tweezers under a binocular stereoscope and subsequently washed in sterile ddH_2_O. Small proportions of these (equivalent to the size of three sand grains) were finally picked off the cleaned lichen fragments and transferred to 200 µl PCR tubes filled with lysis buffer of the Platinum Direct PCR Universal Master Mix - Kit (Thermo Fisher Scientific Inc). Lysis was carried out according to standard protocol provided by the manufacturer and acted as template for subsequent PCRs.

### ﻿Loci amplification of gene regions

Multiple taxonomic gene regions for all studied lichens were selected and amplified based on the latest phylogenetic-based studies available for each lichen genus as outlined in detail below. All PCR reactions were carried out using the Platinum Direct PCR Universal Master Mix - Kit (Thermo Fisher Scientific Inc) in a Mini Amp Plus Thermal Cycler (Applied Biosystems, Thermo Fisher Scientific, Waltham, USA).

- *Heterodermia* (ITS-mtSSU-nuLR): a three locus dataset was created as described by Kondratyuk et al. (2021). The internal transcribed spacer gene region (ITS) was covered by the primers ITS4 and ITS5, the mitochondrial small subunit gene region (mtSSU) was amplified using the primers mrSSU1 and mrSSU3R and the nuLR gene region with the primers LR0R and LR5 and with the PCR conditions as outlined in Suppl. material 1.
- *Cenozosia* (ITS-RPB1-RPB2): a three locus dataset was achieved based on the phylogenetic reconstruction of Spjut et al. (2020). In detail, the ITS gene region was amplified using the primers ITS1 and LR3, the protein coding gene regions of the largest subunit of RNA polymerase II (RPB1) and the second largest subunit of RNA polymerase II (RPB2) with the primer pairs RPB1-VJAFasc, RPB1-VH6R and fRPB2-5F and fRPB2-7CR respectively and the PCR conditions as given in Suppl. material 1.
- *Roccellinastrum* (mtSSU-ITS-RPB1-RPB2): four gene regions were amplified such as the mtSSU, RPB1 and RPB2 as described above for *Heterodermia* and the ITS gene region as outlined above for *Cenozosia* (Suppl. material 1).
- Photobionts: the 18S rDNA gene region for the green algal photobionts of all lichens were amplified using the primers LR3 and Al1500af with details for the primers and PCR conditions given in Suppl. material 1.

All PCR products were checked by gel electrophoresis using 1% E-gel EX (Invitrogen, Thermo Fisher, Waltham, USA) in an E-Gel Power Snap instrument (Invitrogen, Thermo Fisher, Waltham, USA).

Successful PCR products were purified using the NucleoSpin Gel and PCR Clean-up Kit (Marchery Nagel, New England, Canada) according to the manufacturer’s standard protocol. Subsequently, they were sent for Sanger sequencing carried out by Azenta (Göttingen, Germany) with the primers that were for the amplification of the respective gene regions. All generated DNA sequences were deposited at the National Center for Biotechnology Information (NCBI) GenBank.

### ﻿Molecular phylogenetic analyses

Sequences for individual loci were checked against the NCBI GenBank dataset using the Basic Local Alignment Search Tool (BLAST) of Mega 11 (Tamura et al. 2021). Subsequently, multiple sequence alignments were performed using the multiple sequence alignment method (Muscle) algorithm (Edgar 2004), and were carefully checked by eye and manually adjusted. For each dataset, ambiguous alignment sites and intronic regions within RPB1 and RPB2 were manually removed or adjusted when present. Prior to concatenation and for each dataset the evolutionary model that was best suited to the database used was selected on the basis of the lowest Akaike Information Criterion (AIC) value and calculated in Mega X. The phylogenetic trees were calculated based on 1,000 Bootstrap replications and inspected for incongruence.

Significant conflict was detected for *Roccellinastrum* and thus the alignments were not concatenated for this lichen. Instead the single phylogenetic trees were kept including 1162 base pairs of the ITS gene region, 1121 of the protein coding gene region RPB1 and 1115 base pairs of RPB2 as well as 822 bp of the mtSSU gene region (GTR+G+I substitution model). Besides Maximum Likelihood analyses (ML) also Bayesian Inference (BI) was calculated for each of these trees using Mr. Bayes 3.2.1 (Ronquist and Huelsenbeck 2003). In addition, a phylogenetic tree of the ITS region of the two *Heterodermia* specimens was created as described above in order to demonstrate the conflict raised for the rejected split in the genera (Kondratyuk et al. 2021; de Souza et al. 2022).

All other alignments were concatenated so that the final alignment for *Heterodermia* (ITS-mtSSU-nuLR; Suppl. material 2) consisted of 2344 bp complementing the dataset generated by Kondratyuk et al. (2021) while the alignment for *Cenozosia* (ITS-RPB1-RPB2; Suppl. material 3) was made of 2195 bp complementing the dataset given by Spjut et al. (2020) using the GTR+G substitution model for both lichen datasets.

The alignment of the 18S rDNA covering 2398 bp from the photobionts was curated as described above and calculated using the TN93+G substitution model with 1,000 replications for the ML analyses.

Phylogenetic trees were finally depicted in iTOL (Letunic and Bork 2021) and the resulting figures were edited in BioRender or Inkscape.

## ﻿Results

Based on the holistic approach applied here, combining multi loci gene phylogenies, intensive morphological- and chemical analyses, the lichens *Roccellinastrumspongoideum* and *Heterodermiafollmannii* were characterized in addition to the novel species *Heterodermiaadunca*, *Cenozosiaexcorticata* and *C.cava*, which were described here. In addition, information on the lichen’s photobionts could be gained based on morphological and phylogenetic investigations.

### ﻿Morphological characterization and spot tests

**Figure 1.**
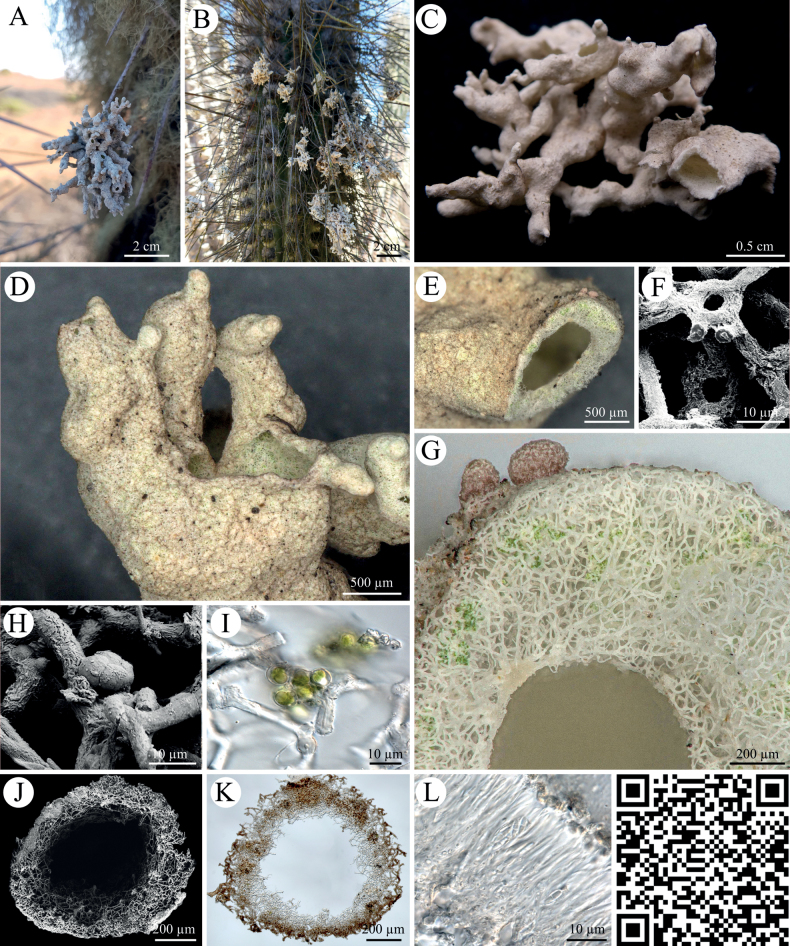
*Roccellinastrumspongoideum***A, B** photographs showing *R.spongoideum* on the downfacing cactus needles of different *Eulychnia* cacti in the National Park Pan de Azucar **C** photograph of byssoid lichen thallus **D, E** close-up of byssoid thallus showing the coarse hyphal structure **F** SEM image of hyphal loops **G** close-up of a cross section with a loose hyphal network, patchy arrangement of photobionts and pink apothecia **H** SEM image of four ‘micareoid’ photobionts on fungal hyphae **I** light microscopy of micareoid photobionts with large vacuole-like structures **J, K** SEM image and light microscopy of the lichen thallus **L** microscopic cross section through apothecium with two-celled spores divided by a septum. QR code redirects to 3D scan of *R.spongoideum*. PW: Lichen.

Thalli of *Roccellinastrumspongoideum* (Fig. 1) were found to be 2 cm large but specimens up to 7 cm were observed with a gray byssoid or tubular thallus hanging down from the needles of cacti of the genus *Eulychnia* spp. Young thalli grow first as erected tufts developing into hollow tubes covered with white to light pink or brownish apothecia. The lichen’s internal structure was made of dichotomously branching, thick hyphae which form a wide and loose thallus mesh with single photobiont nests. In the spot tests, *Roccellinastrumspongoideum* was negative for UV, K and C, but positive for P. Here, the lichen turned yellow/orange. During the KC test, an orange reaction was detected.

**Figure 2.**
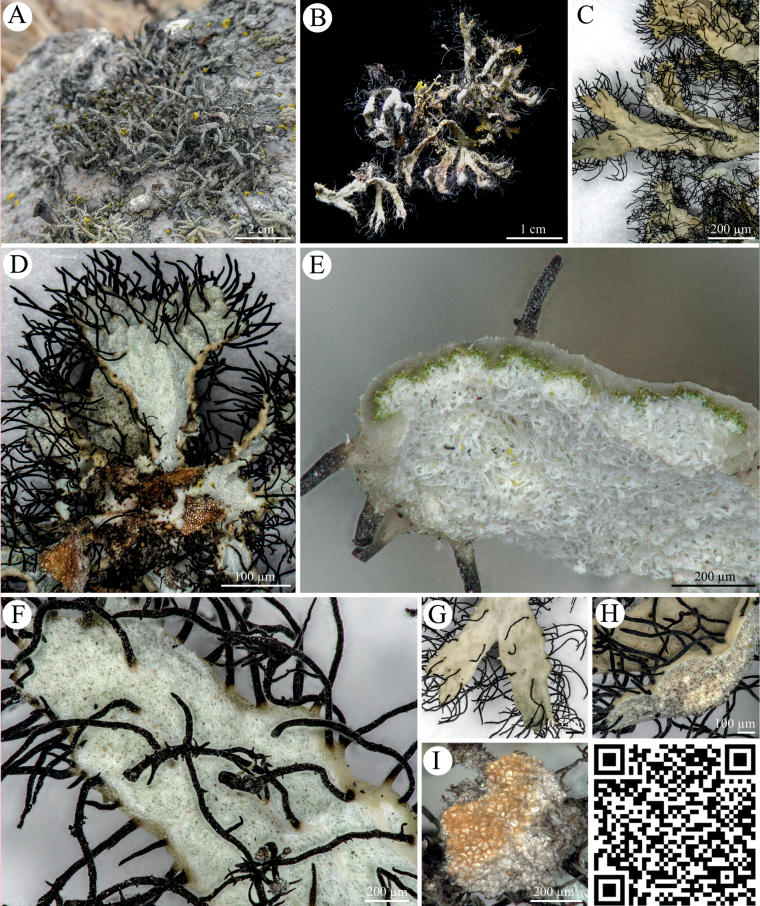
*Heterodermiafollmannii***A, B** photographs of *Heterodermiafollmannii* with its gray thallus and black cilia **C** top view of thallus lobes **D** bottom view of thallus lobes without lower cortex showing the white, loose medulla and brownish attachment sides **E** cross section of thallus lobe showing the photobiont layer and the gray upper cortex which forms a rim on the ventral side with an open medulla **F** ventral side of thallus lobe with open medulla and black, long and branched cilia **G** top view **H** upwards bent terminal thallus part which is slightly bloated **I** ventral view of the attachment side. QR code redirects to 3D scan of *Heterodermiafollmannii*. PW: Lichen.

The gray *Heterodermiafollmannii* (Fig. 2) was mostly found growing on stones or on the lower third of cacti only in a narrow strip close to the coastal cliffs where it formed a centrally attached thallus that divided radially into broader branches. Those branches were often ascending and bullate and covered in a dense set of black cilia. Thalli were found to have an upper cortex that formed swollen thallus margins and an open thallus on the ventral sites. Photobionts were arranged in a typical layer structure. Spot tests showed that *Heterodermiafollmannii* reacted positively to K with a yellow coloration; it was also positive for the KC test with an orange reaction but it did not react to UV, C and P.

**Figure 3.**
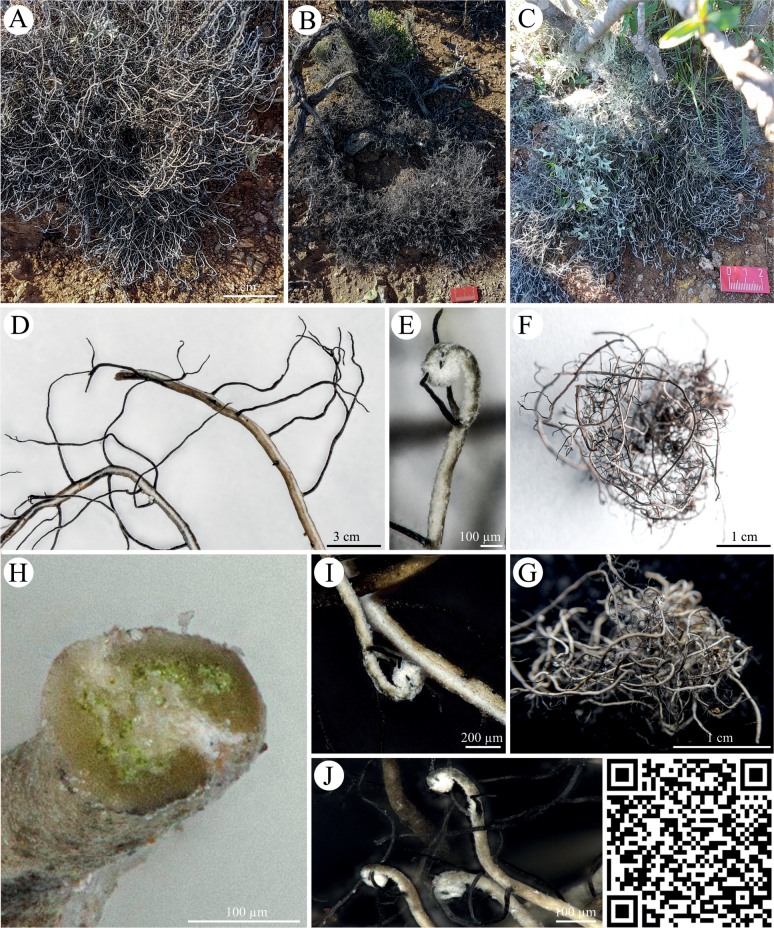
*Heterodermiaadunca* sp. nov. **A–C** photographs of *Heterodermiaadunca* sp. nov. on fine soil in the National Park Pan de Azucar **D** thallus fragments with black cilia and whitish ventral thallus parts forming a rim without lower cortex **E** terminal wrapped thallus part with blackish cilia **F, G** photographs **H** detail of thallus cross section with photobiont layer showing the curved upper cortex that forms a rim where the white medulla is without a lower cortex **I, J** terminal thallus parts with the typical wrapped ends. QR code redirects to 3D scan of *Heterodermiaadunca* sp. nov. PW: Lichen.

*Heterodermiaadunca* sp. nov. (Fig. 3) was characterized by larger tufts formed mostly on the ground or close to cacti stems where it can easily be recognized due to its hairy appearance and almost rounded, thin thallus branches that terminally form coiled hooks. Similar to *L.follmannii*, this species had an upper cortex that formed a cortex lacking rim at ventral sides of the branches. In addition, it was densely covered with black cilia and had a photobiont layer. All spot tests were negative, but the K test showed a slight tendency to a yellow reaction.

**Figure 4.**
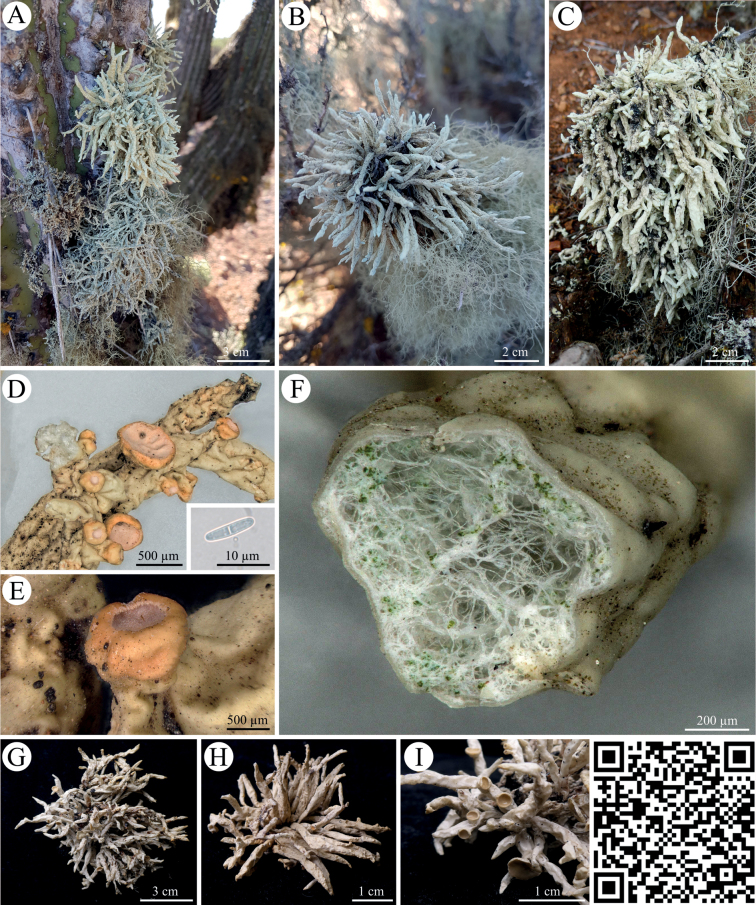
*Cenozosiacava* sp. nov. **A–C** photographs of *Cenozosiacava sp. nov.* on various cacti in the National Park Pan de Azucar **D, E** close-up of light pink apothecia including microscopic image of the two-celled spore with a septum in **D** note the tremelloid, gall-like structures across the thalli **F** close-up of lichen thallus cross section depicting the hollow structure of the thallus, the irregular photobiont nests and the smooth outer cortex structure **G–I** photographs of various thalli and apothecia. QR code redirects to 3D scan of *Cenozosiacava sp. nov.* PW: Lichen.

*Cenozosiacava* sp. nov. was found on cacti (mostly *Eulychnia* spp.) in the fog zones resembling a habitus comparable to *Nieblaceruchis* but with less tapered, hollow thallus parts (Fig. 4). Thalli were white to gray and strongly wrinkled or folded in the dry state but gray-green and significantly less wrinkled if hydrated. The thalli were found to be divided into many long, uniformly narrow cylindrical-teretiform branches that showed only a weakly developed inner hyphal network with isolated photobiont nests.

**Figure 5.**
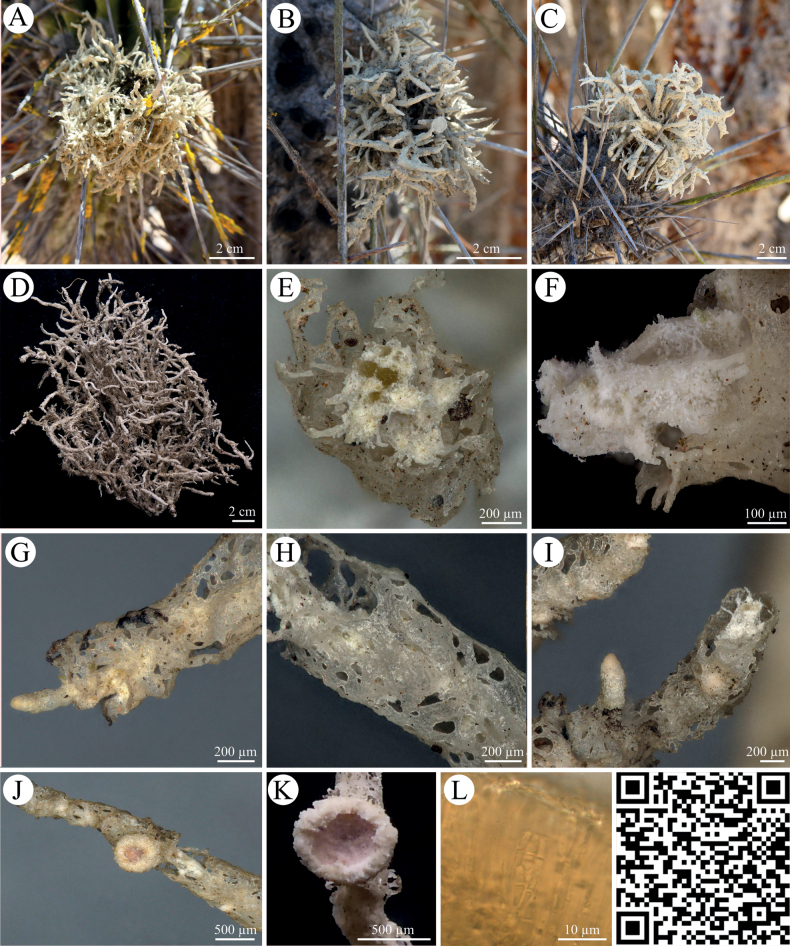
*Cenozosiaexcorticata* sp. nov. **A–D** photographs of *Cenozosiaexcorticata* sp. nov. on cacti needles in the National Park Pan de Azucar and under laboratory conditions **E–I** different close-ups of thallus sections showing the bright white central strand surrounded by a layer of gray, hyaline coalesced hyphae forming a perforated cortex and chondroid strands that crisscross the medulla. Note that new growth of branches is initiated by the whitish inner strand that breaks through the perforated cortex structures (**G, I**) **J, K** apothecia with pink hymenial disc **L** microscopic cross section through the apothecium with two-celled spores divided by a septum. QR code redirects to 3D scan of *Cenozosiaexcorticata* sp. nov. PW: Lichen.

In contrast to *C.cava* sp. nov., *Cenozosiaexcorticata* sp. nov. was characterized by thallus branches that had a unique cortex structure made hyalin, coalesced hyphae that formed a strongly perforated and wide sleeve around the white medulla pillowed by a few hyphal strands that crisscrossed between medulla and cortex sleeve (Fig. 5). This gave an overall chondroid character to the habitus of *C.excorticata* sp. nov. which also showed loose photobiont nests distributed across the outer cortex and the inner medulla strand. The results of the spot tests were similar between the two *Cenozosia* species. Both were negative for UV, K and P. Only for C a color change to red was detectable. For *Cenozosiacava* sp. nov. the complete thallus turned red, while in *Cenozosiaexcorticata* sp. nov. only the central strand turned red, the outer loose thallus remained unstained. The latter also reacted yellow to KC.

### ﻿Chemical characterization

Thin layer chromatography (TLC) revealed that *Roccellinastrumspongoideum* showed atranorin and protocetric acid as secondary lichen substances including two other unknown substances. *Heterodermiafollmannii* and *Heterodermiaadunca* sp. nov. both contained atranorin and zeorin as secondary lichen substances. *Cenozosiacava* sp. nov. and *C.excorticata* sp. nov. were positive for zeorin, decarboxynorstenosporic acid and decarboxydivaricatic acid, in addition to fatty acids. Two other unknown secondary lichen substances were found in *C.cava* sp. nov., and another unknown secondary metabolite was found in *C.excorticata* sp. nov.

### ﻿Molecular characterization

**Figure 6.**
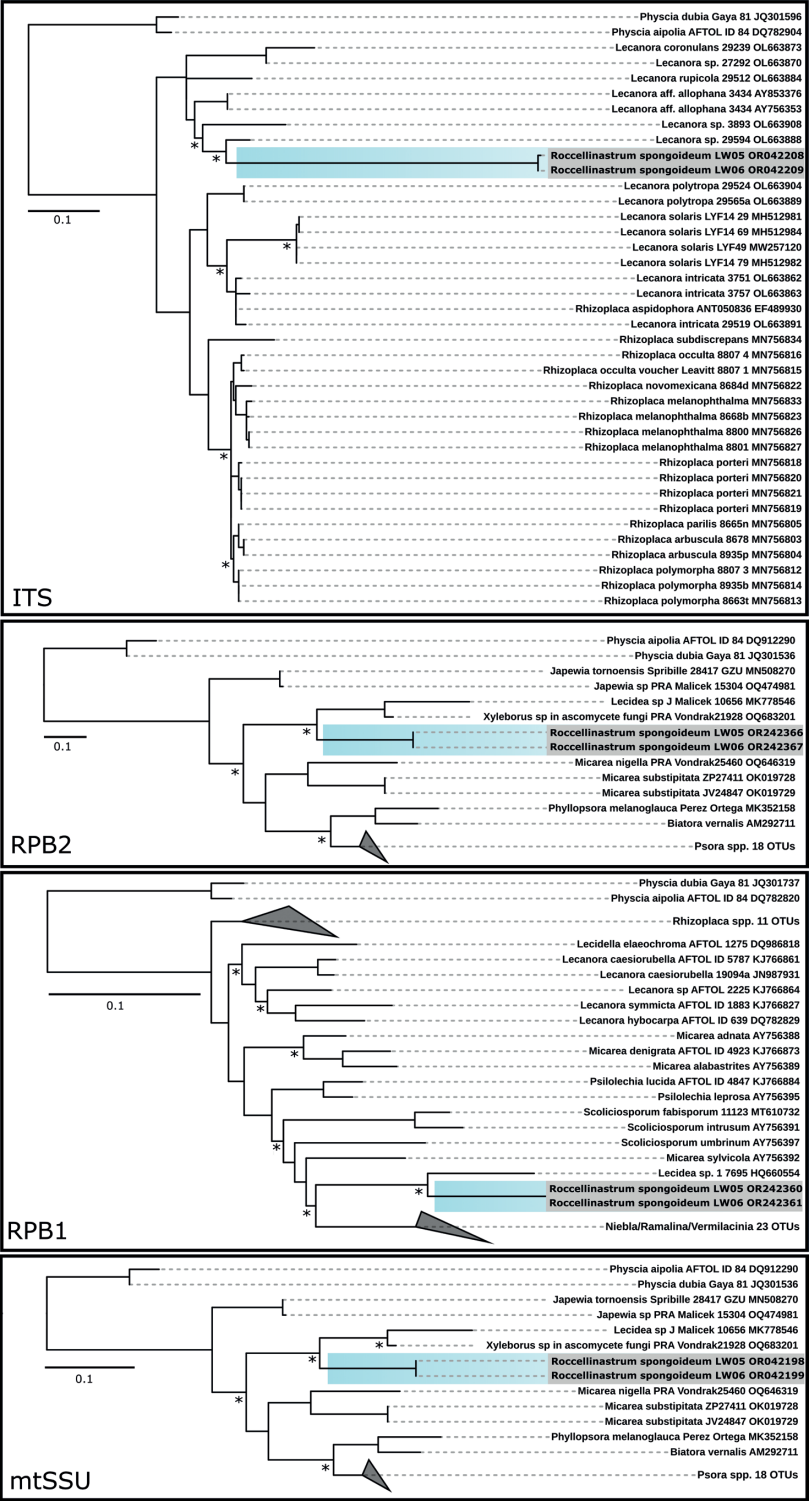
Phylogenetic trees of the ITS, rpb1, rpb2 and mtSSU gene regions of *Roccellinastrumspongoideum*. ML and BI phylogeny with bootstrap values ≥ 80 are marked with an asterisk.

For *Roccellinastrumspongoideum* a significant conflict was detected during the attempt to concatenate the single gene alignments and thus the alignments were not concatenated (Fig. 6). Concerning the ITS gene region it formed a separated cluster within some *Lecanora* sequences. The RPB1 sequences were positioned with a *Lecidea* sequence, while RPB2 and mtSSU were both placed between sequences of *Micareasubstipitata* and *M.nigella* and a *Xyleborus* sp. sequence. For none of the gene regions a relatedness to other sequences greater than 93% could be found.

**Figure 7.**
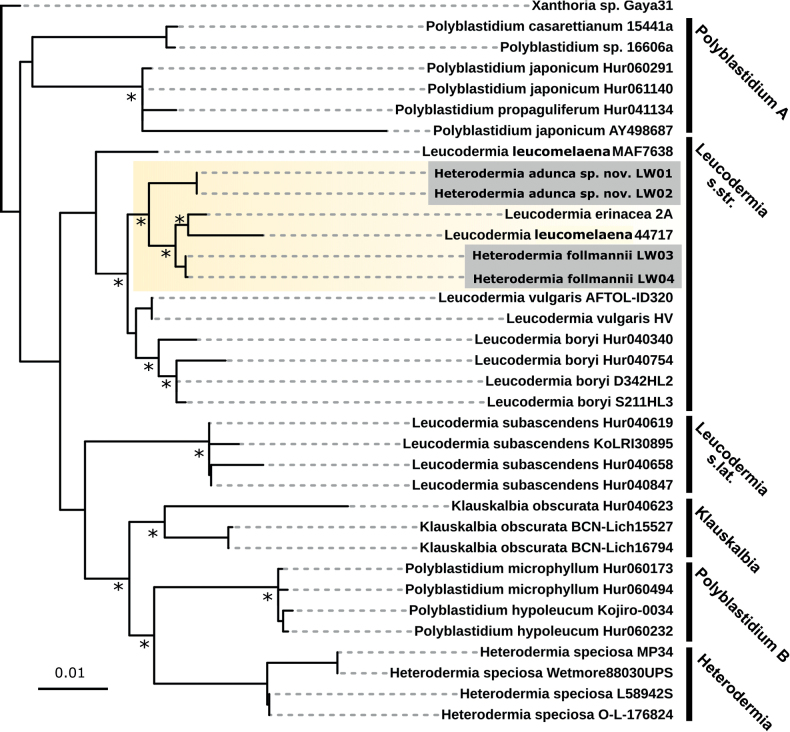
Phylogenetic tree of the concatenated ITS-mtSSU-nuLR gene regions for *Heterodermiaadunca* sp. nov. and *H.follmannii* after Kondratyuk et al. (2021). ML and BI phylogeny with bootstrap values ≥ 80 are marked with an asterisk.

**Figure 8.**
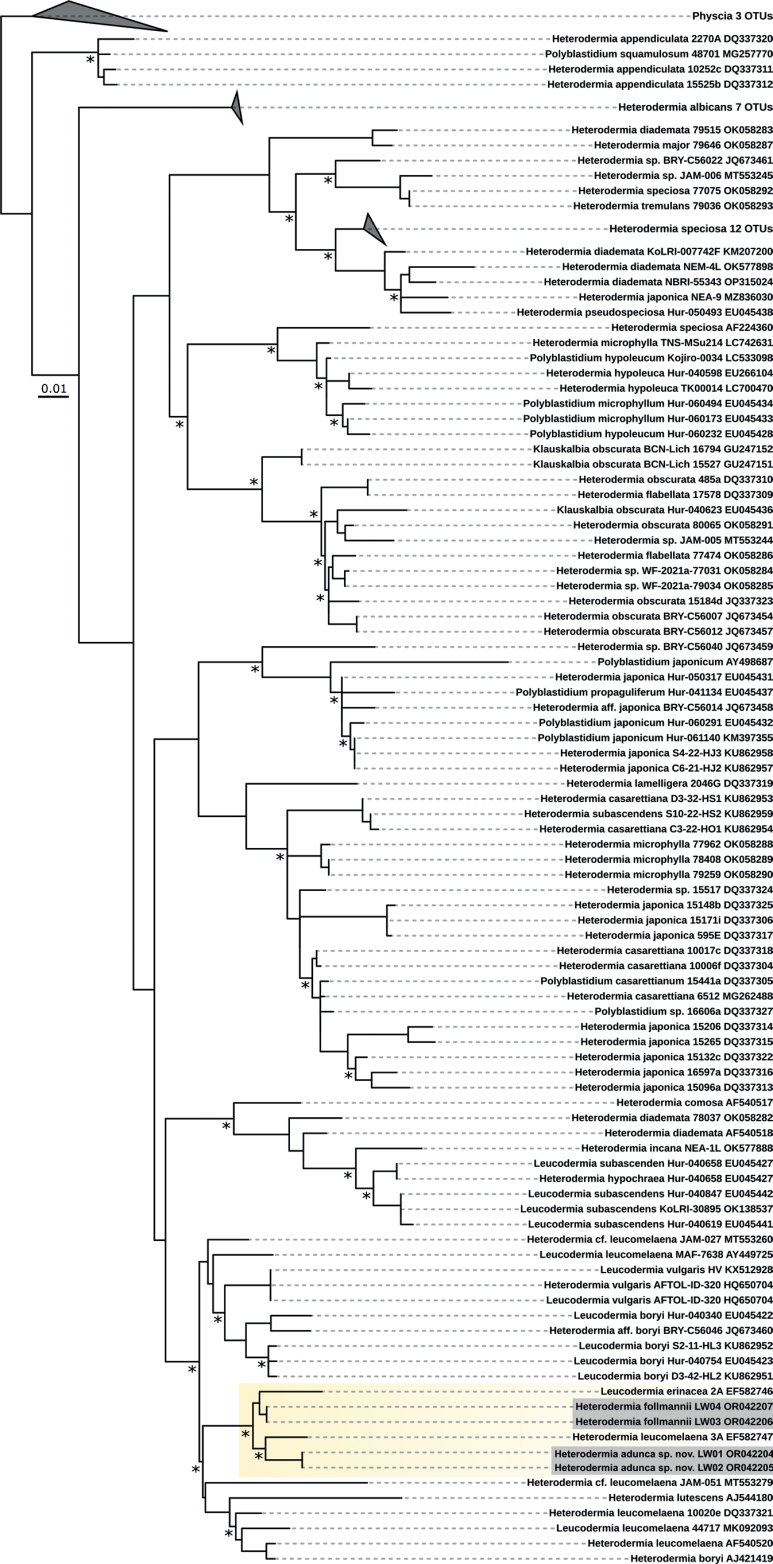
Phylogenetic tree of the ITS gene region for *Heterodermiaadunca* sp. nov. and *H.follmannii* following de Souza et al. (2022). ML and BI phylogeny with bootstrap values ≥ 80 are marked with an asterisk.

For both *Heterodermia* lichens a three loci dataset could be generated covering the ITS, mtSSU and nuLR gene regions (Fig. 7). Neither lichen fell into the *Heterodermia* sensu stricto cluster following Kondratyuk et al. (2021) but well into *Leucodermia* and were also distinct from the *Polyblastidium* branch. In addition, *H.follmannii* and *H.adunca* sp. nov. were separated by *Leucodermiaerinacea* 2A and *L.leucomelaena* 44717 and formed a distinct larger cluster with other *Leucodermia* species which together were split from a set of *L.subascendens* sequences. However, the ITS-only phylogenetic tree (Fig. 8) included many more ITS sequences from *Heterodermia* specimens and represents the latest update to the genus according to de Souza et al. (2022). From this analyses it can be drawn that *H.follmannii* and *H.adunca* sp. nov . formed a well-supported and separated cluster together with one ITS sequence from *H.leucomelaena* 3A and one sequence of *H.erinacea* 2A (Fig. 8).

**Figure 9.**
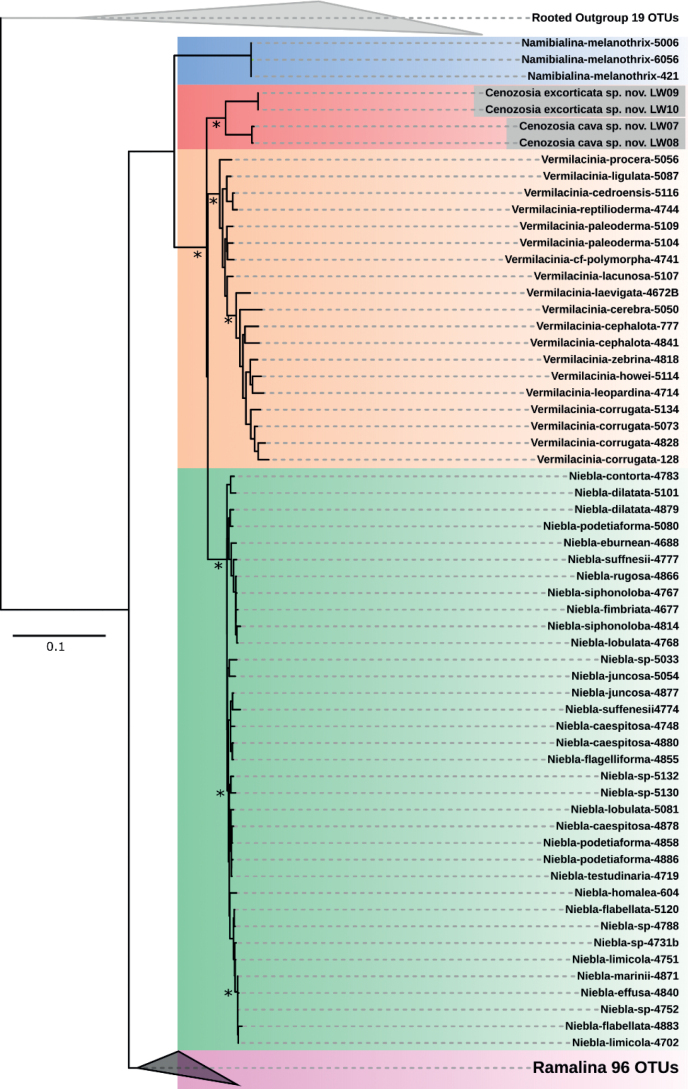
Phylogenetic tree of the concatenated ITS-RPB1-RPB2 gene regions for *Cenozosiaexcorticata* sp. nov. and *C.cava* sp. nov. ML and BI phylogeny with bootstrap values ≥ 80 are marked with an asterisk.

The three gene phylogeny including the ITS, RPB1 and RPB2 gene regions for the *Cenozosia* lichens was created based on the dataset provided by Spjut et al. (2020) and showed a well- supported cluster between *Namibialina* and *Vermilacinia* (Fig. 9).

**Figure 10.**
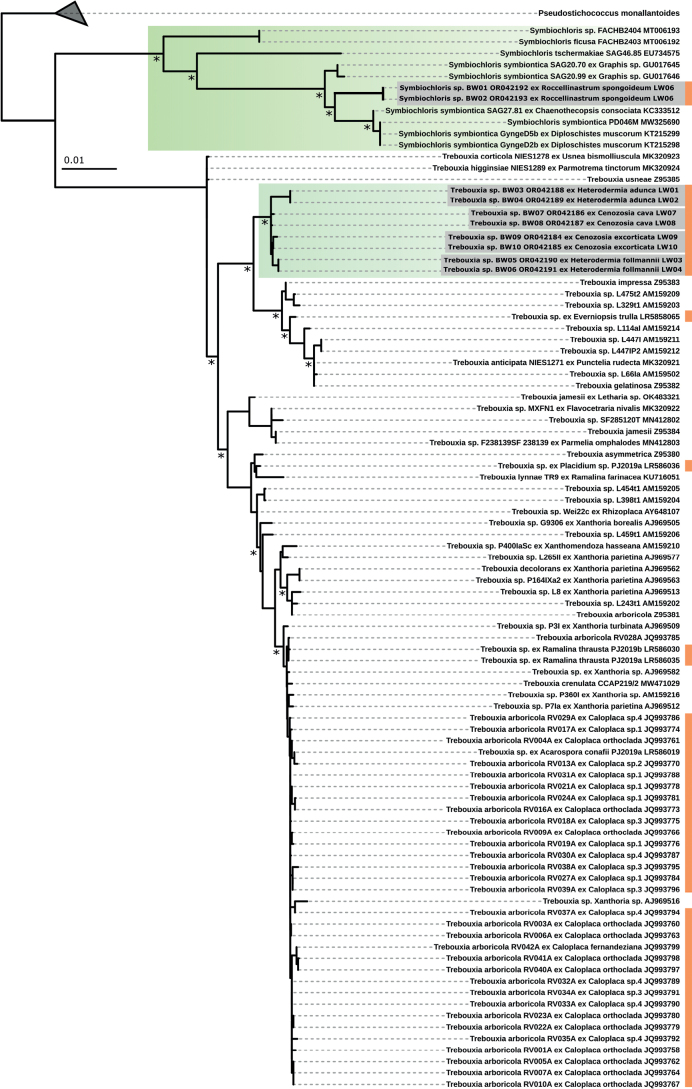
Phylogenetic tree of the 18S rDNA gene region of the lichen photobionts. ML and BI phylogeny with bootstrap values ≥ 80 are marked with an asterisk. Orange bars indicate photobionts of lichens from the Atacama Desert.

The 18S rDNA sequences which were obtained from the photobionts of all five lichens showed that an undescribed *Symbiochloris* species was the photobiont of *Roccellinastrumspongoideum* (Fig. 10), while all other lichens shared trebouxioid algae which formed a well-supported and distinct cluster. This unique cluster was related to the *Trebouxiagelatinosa*/*T.impressaclade* (Fig. 10).

## ﻿Discussion

Fog zones of coastal areas such as the Coastal Range of the Atacama or the Namib Desert have long been proposed as lichen hot spots with a high diversity (Rundel 1978; Lalley and Viles 2005; De los Rios et al. 2022), linked to unique ecophysiological traits of these lichens (Lange et al. 1994; Vondrak and Kubásek 2013; Jung et al. 2019). However, most of them are endemic, such as members of the genus *Santessonia* (Sérusiaux and Wessels 1984), *Lecanographaazurea*, *Roccellinaochracea* (Follmann 2008), *Parmeliahueana* (Büdel and Wessels 1986) or the genus *Camanchaca* (Follmann and Peine 1999) and have been described solely based on morphological investigations. Our study investigated five of the most common and conspicuous lichens of the fog oasis Las Lomitas in the National Park Pan de Azúcar (personal observation) of the coastal Atacama Desert. Although the two species *Roccellinastrumspongoideum* and *Heterodermiafollmannii* were already described (Follmann 1967; Sipman 1995), we finally created a molecular background for these two lichens covering multiple taxonomic gene regions, confirmed the genus *Cenozosia*, described the three new species *C.cava*, *C.excorticata* and *Heterodermiaadunca* and also investigated the green algal photobionts of all five studied lichens. In the following section each lichen species, as well as their photobionts, will be discussed.

### ﻿The byssoid lichen *Roccellinastrumspongoideum*

Due to a byssoid thallus, the epiphytic genus *Roccellinastrum* comprises a set of morphologically conspicuous species with *R.spongoideum* from the Atacama Desert, *R.epiphyllum* from Southern Chile, *R.candidum* from South America, *R.neglectum* from the needles of coniferous trees of New Zealand, and R.lagarostrobi as well as *R.flavescens* from Tasmania, all of which are deemed to be endemic (Henssen et al. 1983; Kantvilas 1996). We have created the first molecular dataset for this genus based on *R.spongoideum* from the Atacama covering the taxonomically relevant gene regions ITS, RPB1, RPB2 and mtSSU (Fig. 6). Although a concatenated phylogeny could not be reached due to missing genetic data from highly related lichen genera, the presented data will allow a comparison of the phylogeny of *R.spongoideum* with all other species of the genus in future. Previously, the genus *Roccellinastrum* was assigned to the Micareaceae based on morphological characters (Eriksson and Hawksworth 1993), but Andersen and Ekman showed in 2004, that the Micareaceae in this circumscription belongs in the Lecanorales, but that it is not monophyletic, which can also be confirmed by this study. In addition, our investigation included a detailed morphological and chemical characterization involving modern techniques so that the lichen substances and spot tests confirmed the results previously reported for *R.spongoideum* (Suppl. material 4). The species can easily be distinguished from the other species of the genus *Roccellinastrum* by the well-developed grayish thallus composed of rather compact, tubular lobes and by the one-septate spores. Most importantly, *R.spongoideum* has so far only been reported on cacti in fog zones along the coast of northern Chile where it represents a very conspicuous lichen (compared with the many and highly abundant epiphytic *Ramalina* and *Usnea* species) hanging down from the needles of cacti. Also, we could confirm the loose hyphal network with its nested photobionts which we visualized in detail (Fig. 1). The latter could be clearly assigned to the green algal genus *Symbiochloris* (Fig. 10), so that the mysterious term ‘micareoid photobiont’ introduced by Coppins in 1983 can finally receive a carefully evaluated and comprehensive meaning.

### ﻿The ciliated lichens *Heterodermiafollmannii* and *H.adunca* sp. nov.

Members of the *Heterodermia*-complex (Physciaceae) can easily be recognized based on cilia that cover their lobed, grayish to white thalli and among foliose lichens they are one of the most common lichens in tropical and subtropical regions, with a few species reaching temperate regions. Many ‘*Heterodermia*’ species have been described from North America, India, Africa, Europe, Thailand and many other regions (Moberg 2011; Mongkolsuk et al. 2015). However, only recently this *Heterodermia*-complex was investigated based on a multi-gen phylogenetic approach so that *Heterodermia* s.lat. comprised members of the genera *Heterodermia* s.str., *Klauskalbia*, *Leucodermia* and *Polyblastidium* (Kondratyuk et al. 2021). We used the same gene regions for the two lichen specimens from the Atacama Desert and could confirm not only the monophyly of *Leucodermia* and the polyphyly of *Polyblastidium* (Fig. 7) which was previously stated by the authors, but also found that the Atacama specimens clustered in the well supported *Leucodermia* genus *sensu*Kondratyuk et al. (2021) (Fig. 7). In contrast to this and according to the latest update for the *Heterodermia*-complex introduced by de Souza et al. in 2022 the above described split of the genus has been rejected based on a large phylogenetic set comprising ITS sequences. For this reason, we also created a large ITS phylogeny (Fig. 8) which supported these results, showing no evidence for the split introduced by Kondratyuk et al. (2021). Interestingly, the position of the ITS sequences of *H.adunca* sp. nov. and *H.follmannii* remained similar to the three-locus dataset: they formed a separated, well-supported cluster with ITS sequences from *Heterodermialeucomelaena* 3A and *H.erinacea* 2A (Fig. 8). As the split of the genera has been rejected by de Souza et al. (2022) we here also assigned the two investigated specimens to the genus *Heterodermia* and not to *Leucodermia* as one would draw from the three-locus phylogeny (Fig. 7) reproduced from Kondratyuk et al. (2021). The assignment of the two lichens to *Heterodermia* was also confirmed by the presence of atranorin and zeorin as secondary lichen substances, a trait shared among all of these genera (Suppl. material 4). As already expected based on their highly differing morphology, the two investigated lichens specimens showed divergent positions within the *Heterodermia* cluster: While the specimen morphologically resembling *Heterodermiafollmannii* (Follmann 1967; Sipman 1995) was characterized by curved, densely ciliated thallus branches (Fig. 2), the new species *H.adunca* was characterized by almost rounded, thin branches with a terminal, helix-like hook (Fig. 3). However, the latter might be confused with *Heterodermiacircinalis* but this species has its main distribution in the paramos of South America, above 3.000 m (e.g. Mongkolsuk et al. 2015) which indicates a significantly different ecology compared to the Coastal Range of the Atacama Desert. In addition, *H.circinalis* is P+ in both, the medulla and the cortex and has leucotylin (e.g. Mongkolsuk et al. 2015; Kurokawa 1962) whereas *H.adunca* was P- in all parts and did not contain leucotylin.

### ﻿*Cenozosia*, the Atacama sister genus of *Niebla*, *Ramalina*, *Namibialina* and *Vermilacinia*

The Ramalinaceae is the fourth-largest family of lichenized ascomycetes and their fruticose genera were recently updated and now support the four genera *Ramalina* (sub-cosmopolitan), *Namibialina* (endemic to South West Africa), *Niebla* and *Vermilacinia* (endemic to the New World) (Spjut et al. 2020). Most of these genera occur in fog-influenced, arid desert environments and also members of the genus *Niebla* were frequently reported from the coastal Atacama Desert (Rundel 1978; Harańczyk et al. 2021). However, already in 1981, Bowler noted that the species *Cenozosiainanis* - the type of the genus *Cenozosia* - described as an endemic species from the Atacama Desert resembles *Nieblaceruchis* (Bowler, 1981), probably caused by a similar macroscopic thallus structure. Later, more detailed morphological investigations carried out by Spjut (1996) indicated that *C.inanis* is characterized by a perforated cortex and chondroid strands that crisscross the medulla. Further on, in 2018, Kistenich et al. generated a short LSU sequence of *C.inanis* from the Atacama Desert and stated it as sister to *Cliostomum* and the other fruticose taxa now recognized in the Ramalinaceae. These results could not be supported by Spjut et al. (2020) who investigated this complex based on a four locus gene phylogeny. Moreover, they expected the genus *Cenozosia* to be resolved at the base of the clade formed by *Vermilacinia* and *Niebla*, but themselves regretted that they could not include lichen specimens from the Atacama Desert. Our results now supported the observation of a unique cortex structure (Fig. 4, 5), a hollow thallus that crisscrosses the medulla (Fig. 4, 5) and we resolved the genus *Cenozosia* as the missing member of the Ramalinaceae endemic to South America in a well-supported cluster using a three-locus phylogeny set (Fig. 9). Also the secondary metabolites decarboxynorstenosporic acid and decarboxydivaricatic acid that we detected in both investigated *Cenozosia* species were so far not reported for any species of the genus *Ramalina*, *Niebla*, *Namibialina* or *Vermilacinia* (Suppl. material 4, Spjut et al. 2020). Finally, we described the two new species *C.cava* and *C.excorticata* which both significantly differed from the herbarium specimen of *C.inanis* [TROM-L-45999]. Interestingly, both species described by us could easily be differentiated based on their thallus structures: while *C.cava* sp. nov. showed a thallus habitus similar to *Nieblaceruchis* and *C.inanis* with many long, uniformly narrow cylindrical-teretiform, flexuous and hollow branches (Fig. 4), *C.excorticata* sp. nov. was characterized by a fenestrate outer cortex and an inner, white medulla strand (Fig. 5). As an addition to the identification key introduced by Spjut in 1996, the genus *Cenozosia* is morphologically similar to members of the genus *Niebla* sharing the feature of chondroid strands. *Cenozosiacava* can be confused with *Nieblaceruchis* but can be differentiated by the smaller, less pronounced formation of apothecia. In contrast, *C.excorticata* has the unique feature of the fiber-like outer cortex which forms a loose mesh surrounding the inner, whitish strand. Currently, it is difficult to present a full addition to the key of Spjut from 1996 because only the two Cenozosia species presented here have yet been evaluated. Those two species have a very distinct morphology compared with each other so that a detailed set of features framing the genus *Cenozosia* needs to be created in future based on additional members of the genus. This will verify if uniform morphological features such as hollow thallus branches or a fenestrate outer cortex or even different characteristics best characterize the genus, a step we cannot go here. However, together with *C.inanis* the two described species *C.cava* sp. nov. and *C.excorticata* sp. nov. are currently the only members of the genus that have been investigated and which share the habitat of the coastal Atacama Desert. *Niebla* species have been detected in the Atacama Desert but these observations were solely based on morphological investigations and it remains open, if members of the genus *Niebla* can be found at this habitat at all, or if these lichens must be assigned to the genus *Cenozosia* after molecular analyses.

### ﻿The lichen’s photobionts

It has often been assumed that lichens ehich share a unique ecological niche such as those occurring in a fog oasis also share a common set of photobionts determining the ecophysiological potential of those lichens. In 2020, a multi-locus DNA backbone helped to provide a framework for the identification of trebouxioid lichen photobionts (Muggia et al. 2020) which will now help to tackle this idea. Only a small set of lichens from the Atacama Desert photobionts have been investigated based on DNA sequences or cultures (e.g. Castillo and Beck 2012; Jung et al. 2019), which could now be augmented by our results. Including the aforementioned DNA sequences, we found a unique *Trebouxia* cluster related to the *T.gelatinosa*/*T.impressa* lineage *sensu*Muggia et al. (2020) made of photobionts from *Cenozosiacava* sp. nov., *C.excorticata* sp. nov., *Heterodermiafollmannii* and *H.adunca* sp. nov. (Fig. 10), that is significantly different to Trebouxia photobionts found for *Ramalinathrausta*, *Acarsoporaconafii*, *Everniopsistrulla* and *Placidium* sp. based on the investigated gene region (Jung et al. 2019), and the *Caloplaca* photobionts investigated by Castillo and Beck (2012) from the Atacama Desert (Fig. 10, marked in orange), most of which grouped in the *T.arboricola* lineage *sensu*Muggia et al. (2020). This is interesting because the four investigated lichens of this study and those of Jung et al. (2019) shared the same location - the National Park Pan de Azúcar. Moreover, this is also remarkable because the four lichen species treated here do not share the same ecological niche: the two *Heterodermia* species grow on stones and sometimes epiphytically, only in a small strip on the edge of the coastal cliff where dense fog covers appear, coupled with high wind speeds, while both *Cenozosia* species grow abundantly but exclusively epiphytically on cacti also further inland where only sips of fog come in. In addition, *H.follmannii*, *H.adunca* sp. nov. and also other *Heterodermia* species have also been detected in Alto Patache (1.300 km further North from Pan de Azúcar; personal observations), where neither *Cenozosia* nor cacti occur.

A different story unrolls for the photobiont of *R.spongoideum* which was previously characterized as micareoid (Henssen et al. 1983) and now could be assigned to the genus *Symbiochloris* (Fig. 10). This genus encompasses free-living and/or lichenized algae with lobed chloroplasts and that reproduce by forming zoospores with two subapical isokont flagella that emerge symmetrically near the flattened apex (Škaloud et al. 2016). Future studies including molecular phylogenies will show if the term micareoid will always refer to *Symbiochloris* or to *Diplosphaerachodatii* as assumed earlier by Voytsekhovich et al. (2011) solely based on morphological characteristics. As only a few species of *Symbiochloris* have been described it can already be drawn from our phylogeny that the photobiont of *R.spongoideum* represents an unknown, yet to be described species.

### ﻿Taxonomic novelties

#### ﻿ Roccellinastrum spongoideum

Taxon classificationFungiLecanoralesByssolomataceae

(Follmann)

A4F11C33-454D-5AB0-B509-4950BE548273

345795

Fig. 1 after Follmann (1967) , including Henssen et al. (1983) and own observations

##### Description.

Thallus usually 2 cm large but specimens up to 7 cm were observed, gray to brownish gray, sub- fruticose, byssoid-spongiose or cottony-granular. Lobes tubular, up to 20 mm long or longer, cylindrical and up to 5 mm broad or flattened and up to 6 mm broad, partly fenestrate in older parts. Young thalli at first as erect tufts developing to hollow tubes covered with white to light pink or brownish apothecia which are laminal, up to 0.5 mm broad, frequently compound, sessile or shortly stipitate. Hymenium 35–45 µm high, paraphyses branched, hypothecium colorless. Asci 26–35 × 8–10 µm, spores 1-septate at maturity, 7.5–10 × 1.5–3 µm. Excipulum of (often dichotomously) branched, radiating hyphae with strongly gelatinized walls, colorless, not sharply delimited from the paraphyses. Single hyphae often form loop structures in inner thallus parts. Thallus hyphae 3–9 µm wide, lumina ca. 1 µm thick. Cell lumina elongated or short, the ends sometimes characteristically enlarged adjacent to septa. Cells of the *Symbiochloris* photobionts (= ‘micareoid’) of 8–10 µm in diameter, arranged in nests of several cells.

##### Secondary metabolites.

Atranorin, protocetraric acid and traces of two unknown constituents. UV-, K-, C-, KC+ orange, P+ yellow to orange.

##### Distribution and ecology.

The endemic species grows epiphytically on downwards pointing needles mainly of the cactus *Eulychnia* sp. along the Coastal Range of the Atacama Desert of Chile where fog frequently occurs.

##### Notes.

The pale brownish tinge of the apothecia is caused by crystals of pigments deposited on the outer surface of the fruiting bodies. It seems that Follmann (1967) misinterpreted these crystals when he described a dark hypothecium and excipulum; in sections of the apothecium, hypothecium and excipulum are, in fact, colorless. *R.spongoideum* is easily distinguished from the other species by the well-developed grayish thallus composed of rather compact, tubular lobes, by the one-septate spores, and by its restricted habitat on cacti in fog zones along the coast of northern Chile.

##### Specimens examined.

specimen HBG-025791 (Herbarium Hamburgense, Hamburg, Germany) from Chile, Atacama Desert, Pan de Azúcar National Park.

#### ﻿ Heterodermia follmannii

Taxon classificationFungiCalicialesPhysciaceae

(Sipman)

11426BF9-64C4-587A-911A-2705FDA3DB8E

415692

Fig. 2 after Sipman (1995), and own observations

##### Description.

Thalli up to 5 cm, only attached at central parts by whitish to brown rhizines, which are significantly thinner and shorter than the cilia. Thallus divided into linear, ca. 2–4 mm wide, often dichotomously or palmately branched, ascending, often bullate lobes. Upper side flat or slightly convex to concave; lower side decorticate, without rhizines, ca. 0.8–1.3 mm wide, bordered by rather swollen thallus margins, sometimes with weak and irregular cartilaginous ridges. Internodes ca. 1–1.5 mm long. Cilia are conspicuous and dense, ca 3 or more per mm on each side, black, usually up to 5 mm long, mostly simple, occasionally with a few terminal bifurcations or a few perpendicular branchlets. The cilia are not very strictly marginal, and may be implanted more or less away from the margin, on the lower, lateral, or upper side of the margin, or on the upper surface, incidentally also on ridges of the lower side. Soredia present, farinose, produced in slightly greenish or blue greenish soralia on the lower side of upturned lobe tips. Soralia poorly delimited and perhaps covering all of the lower surface of the lobes, but most distinct near the lobe tips. In cross section with a cortical layer of very irregular thickness, forming pronounced longitudinal ridges, ca 25–40 µm thick on thin spots, up to 100–150 µm at the ridges, with ca. 100 µm thick medulla. Apothecia and pycnidia unknown. Trebouxioid photobionts are arranged in a continuous layer.

##### Secondary metabolites.

Atranorin, zeorin, weak traces probably of additional terpenoids. Cortex UV-, C-, K+ yellow, KC-, P-; medulla UV-, C-, K-, P-.

##### Distribution and ecology.

The species grows attached to stones or epiphytically on the lower third of cacti restricted to a small strip on top of the steep coastal ridge with high wind speeds and regular fog events. It is also known from the neighborhood of Iquique (Follmann 1994; Alto Patache personal observation), near the coast in northern Chile. Here it grows on SW-facing slopes between ca. 600–1300 m, on low vegetation or directly on soil, in desert vegetation with increased precipitation by fog. Often appears together with *H.adunca*.

##### Notes.

*Heterodermiafollmannii* is morphologically indistinguishable from *H.multiciliata* except by its lobes with upturned tips and sorediate lower side, and by the absence of apothecia. The great morphological similarity suggests that it is a vegetatively reproducing species derived from *H.multiciliata*. It may be most easily confused with *H.comosa*, which shares the regularly branched, ascending lobes which are sorediate on the lower surface. *H.comosa* differs by its flabellate-expanded, not linear lobes, and its usually white cilia, which are exceptionally darkened at the tips. According to the three-gen phylogenetic reconstruction presented here the species falls within the monophyletic *Leucodermia* cluster and is separated from *H.adunca*. According to the ITS-only phylogeny the species falls in a separated cluster together with *H.adunca*, one *H.leucomelaena* and one *H.erinacea* ITS sequence.

##### Specimen examined.

specimen HBG-025794 (Herbarium Hamburgense, Hamburg, Germany) from Chile, Atacama Desert, Pan de Azúcar National Park.

#### ﻿ Heterodermia adunca

Taxon classificationFungiCalicialesPhysciaceae

Jung & Werner sp. nov.

6123AA4F-A992-51B4-AF8D-8028F16C5C45

848941

Fig. 3

##### Type.

Chile. Atacama Desert, Pan de Azúcar National Park (25°59'03"S, 70°36'55"W; 764 m a.s.l.) specimen HBG-025795 (Herbarium Hamburgense, Hamburg, Germany).

##### Diagnosis.

Recognized by its ‘hairy’ growth of the densely ciliated, thin branches.

##### Etymology.

The epithet ‘*adunca*’ refers to the curled, hooked tips that the species produces regularly.

##### Description.

Thallus appears hairy and is attached at various parts by inconspicuous, small whitish to brown rhizines, which are significantly thinner and shorter than the cilia. Cilia are black, emerging from the upper cortex, sometimes branched, up to 5 mm in length. Thread-like lobes that have a rounded appearance created by the curving around of the upper cortex forming a rim, but the threads are clearly made up of upper and lower parts once investigated with a good hand lens. Upper cortex present, smooth, white to pale gray, parts that are closer to the substrate often appear brownish (mature parts). Lower cortex missing, surface appears rough and whitish between the rims. Main lobes are several cm long and often stretch out horizontally from which irregular side branches emerge that are shorter than the main branches. Tips of the branches are often tightly curled under forming short spirals. Pycnidia and apothecia absent. Trebouxioid photobionts are arranged in a continuous layer.

##### Secondary metabolites.

Atranorin, zeorin. UV-, C-, K+ slightly yellow, KC-, P-.

##### Distribution and ecology.

The species grows attached to stones or on soil restricted to a small strip on top of the steep coastal ridge with high wind speeds and regular fog events. It has also been observed at Alto Patache (Iquique), near the coast in northern Chile. There it grows on SW-facing slopes between ca. 600–1300 m, on low vegetation or directly on soil, in desert vegetation with increased precipitation by fog. Often appears together with *H.follmannii*.

##### Notes.

According to the three-gen phylogenetic reconstruction presented here the species falls within the monophyletic *Leucodermia* cluster and is separated from *H.follmannii*. According to the ITS-only phylogeny the species falls in a separated cluster together with *H.follmannii*, one *H.leucomelaena* and one *H.erinacea* ITS sequence. In addition, the species might be confused with *Heterodermiacircinalis* but the latter has broader, more flat lobes, its main distribution in the paramos of South America, above 3000 m, its medulla and cortex are P+ and the lichen contains leucotylin.

#### ﻿ Cenozosia cava

Taxon classificationFungiLecanoralesRamalinaceae

Jung & Werner sp. nov.

E06B29C0-13D2-5FBD-A3FC-374343ACFD5F

848943

Fig. 4

##### Type.

Chile. Atacama Desert, Pan de Azúcar National Park (25°59'03"S, 70°36'55"W; 764 m a.s.l.) specimen HBG-025793 (Herbarium Hamburgense, Hamburg, Germany).

##### Diagnosis.

Similar to *Nieblaceruchis*, but this species has not been verified from the Atacama Desert. *C.cava* also forms smaller and rarer apothecia.

##### Etymology.

Epithet ‘*cava*’ refers to the hollow thallus.

##### Description.

Thallus white to gray and strongly wrinkled or folded in the dry state, gray-green and significantly less wrinkled if hydrated, divided into many long, uniformly narrow cylindrical-teretiform, flexuous branches from a pale brown to blackened base, up to 7.0 cm long and 0.5 cm thick. Mostly made of primary, fastigiate branches, sometimes dichotomously divided. Cortex present, gray, 40–60 μm thick. Medulla white, very loose, with single hyphal strands crisscrossing the hollow interior of the thalli. Apothecia, round, flat, bowl-shaped when young, pale brown to slightly orange with a pale, concave, pink disc, mostly emerging lateral, sometimes terminal, up to 0.8 cm in diameter. Spores two-celled, divided by a septum. Pycnidia black, forming conspicuous, conical protrusions throughout the thallus. Trebouxioid photobiont arranged in nests throughout the loose medulla network.

##### Secondary metabolites.

Decarboxynorstenosporic acid, decarboxydivaricatic acid, zeorin, fatty acids. UV-, K-, C+ red, KC-, P-.

##### Distribution and ecology.

Epiphytically directly on cacti stems, preferably on *Eulychnia* sp., in the fog zones together with *C.excorticata* and various *Ramalina* species.

##### Notes.

The species is similar to *Nieblaceruchis* but can clearly be differentiated from the latter based on its phylogeny and the smaller, less pronounced formation of apothecia.

#### ﻿ Cenozosia excorticata

Taxon classificationFungiLecanoralesRamalinaceae

Jung & Werner sp. nov.

50946FE8-C2AE-5FBF-AC2A-1C37DB14EAB9

848944

Fig. 5

##### Type.

Chile. Atacama Desert, Pan de Azúcar National Park (25°59'03"S, 70°36'55"W; 764 m a.s.l.) specimen HBG-025792 (Herbarium Hamburgense, Hamburg, Germany).

##### Diagnosis.

Recognized by its perforated, leprose thallus.

##### Etymology.

The epithet ‘*excorticata*’ refers to the chondroid, perforated cortex which is loosely wrapped around a whitish medulla that penetrates the cortex during new growth.

##### Description.

Thallus white to gray-brown with a leprose appearance, forming nest-like structures around 5 cm across but also large examples of more than 12 cm have been observed. Thallus divided into many long branches, up to 9 cm long, 0.3 mm thick, narrow cylindrical-teretiform but terminally kinked with a pale brown to blackened base. Mostly made of primary, fastigiate branches, sometimes dichotomously divided, especially towards the tips. Cortex made of pale, gray, hyaline, coalesced hyphae, forming a strongly perforated and wide sleeve around the white medulla pillowed by a few hyphal strands that crisscross between medulla and cortex sleeve. Medulla white, forming a loose, irregular strand made of crisscrossing hyphae within the cortex sleeve. During new growth the whitish medulla strand penetrates the cortex. Juvenile medulla strands without cortex sleeve are terminally light brown. Apothecia, round, bowl-shaped, gray with a concave, pink disc, mostly emerging lateral, sometimes terminal, up to 0.5 cm in diameter. Spores two celled, divided by a septum. Trebouxioid photobiont arranged in infrequent nests throughout the loose medulla and cortex network.

##### Secondary metabolites.

Decarboxynorstenosporic acid, decarboxydivaricatic acid, zeorin. UV-, K-, C+ red, central strand only, KC+ yellow, central strand only, P-.

##### Distribution and ecology.

Epiphytically directly on cacti stems or needles, preferably on *Eulychnia* sp., in the fog zones together with *C.cava* and various *Ramalina* species.

##### Notes.

Similar to various *Niebla* species but endemic to the Atacama Desert forming the distinct *Cenozosia* cluster.

## ﻿Conclusions

This work sets a new framework for fog zone lichens of the Atacama Desert that can help to pinpoint the identities of related fog zone lichens from other parts of the Atacama or even from other deserts. The generated 3D models will enable lichenologists, as well as the national park rangers or tourists, to easily and correctly identify the presented lichen in the field. We also focused on a detailed morphological description of the investigated lichens so that the rangers of the national park can prepare a concept for e.g. a monitoring approach. Regular lichen counts could help to track the health status of the ecosystem that is influenced by mining activities which cause dust turbulence and air pollution. Some lichens can be negatively influenced by these forms of stresses resulting in a die-back that can now be monitored in the long term. This article has also indicated a potentially new *Trebouxia* photobiont cluster and a new *Symbiochloris* photobiont species. In a follow-up study we aim to isolate these photobionts in order to determine their ecophysiology and to further elucidate their phylogenetic and taxonomic positions.

## Supplementary Material

XML Treatment for Roccellinastrum spongoideum

XML Treatment for Heterodermia follmannii

XML Treatment for Heterodermia adunca

XML Treatment for Cenozosia cava

XML Treatment for Cenozosia excorticata

## ﻿Additional information

### Author ORCIDs

Patrick Jung https://orcid.org/0000-0002-7607-3906

Laura Briegel-Williams https://orcid.org/0000-0003-3926-8840

Dina Emrich https://orcid.org/0000-0002-2490-8545

Michael Lakatos https://orcid.org/0000-0002-2636-8917

### Data availability

All generated sequences were submitted to NCBI and can be found in supplementary material or in the figures.

## Supplementary materials

Supplementary material 1 PCR conditions and primers for each locus Data type molecular (word document) Explanation note PCR conditions and primers for each locus: ITS, LSU, RPB1, RPB2, mtSSU, nuLR, 18S. https://binary.pensoft.net/file/886376This dataset is made available under the Open Database License (http://opendatacommons.org/licenses/odbl/1.0/). The Open Database License (ODbL) is a license agreement intended to allow users to freely share, modify, and use this Dataset while maintaining this same freedom for others, provided that the original source and author(s) are credited.Patrick Jung, Lina Werner, Laura Briegel-Williams, Dina Emrich, Michael Lakatos

Supplementary material 2 Accession numbers used for the concatenated alignment of *Heterodermia* Data type molecular (word document) https://binary.pensoft.net/file/886377This dataset is made available under the Open Database License (http://opendatacommons.org/licenses/odbl/1.0/). The Open Database License (ODbL) is a license agreement intended to allow users to freely share, modify, and use this Dataset while maintaining this same freedom for others, provided that the original source and author(s) are credited.Patrick Jung, Lina Werner, Laura Briegel-Williams, Dina Emrich, Michael Lakatos

Supplementary material 3 Accession numbers used for the concatenated alignment of *Cenozosia* Data type molecular (word document) https://binary.pensoft.net/file/886378This dataset is made available under the Open Database License (http://opendatacommons.org/licenses/odbl/1.0/). The Open Database License (ODbL) is a license agreement intended to allow users to freely share, modify, and use this Dataset while maintaining this same freedom for others, provided that the original source and author(s) are credited.Patrick Jung, Lina Werner, Laura Briegel-Williams, Dina Emrich, Michael Lakatos

Supplementary material 4 Spot tests and TLC Data type chemical tests (word document) Explanation note Secondary metabolites and spot test reactions. https://binary.pensoft.net/file/886379This dataset is made available under the Open Database License (http://opendatacommons.org/licenses/odbl/1.0/). The Open Database License (ODbL) is a license agreement intended to allow users to freely share, modify, and use this Dataset while maintaining this same freedom for others, provided that the original source and author(s) are credited.Patrick Jung, Lina Werner, Laura Briegel-Williams, Dina Emrich, Michael Lakatos
