# Supplementary material for: ﻿Roccellinastrum, Cenozosia and Heterodermia: Ecology and phylogeny of fog lichens and their photobionts from the coastal Atacama Desert
Source: MycoKeys. 2023 Aug 1;98:317–48. doi: 10.3897/mycokeys.98.107764 (PMC10410537; doi:10.3897/mycokeys.98.107764)
Supplement: Supplementary material 1 — PCR conditions and primers for each locus [file mycokeys-98-317-s001.docx]

| Locus | Primer name | Primer sequence (5´-3´) | Cycle conditions | References |
| --- | --- | --- | --- | --- |
| ***Heterodermia*** | | | | |
| ITS | ITS4 | TCCTCCGCTTATTGATATGC | *Platinum*™ *Direct PCR Universal Master Mix*-Kit | White et al. (1990) |
|  | ITS5 | GGAAGTAAAAGTCGTAACAAGG |  |  |
| mtSSU | mrSSU1 | AGCAGTGAGGAATATTGGTC | *Platinum*™ *Direct PCR Universal Master Mix*-Kit | Zoller et al. (1999) |
|  | mrSSU3R | ATGTGGCACGTCTATAGCCC |  |  |
| nuLR | LR0R | ACCCGCTGAACTTAAGC | *Platinum*™ *Direct PCR Universal Master Mix*-Kit | Rehner and Samuels (1994) |
|  | LR5 | ATCCTGAGGGAAACTTC |  | Vigalys and Hester (1990) |
| ***Cenozosia*** | | | | |
| ITS | ITS1f | CTTGGTCATTTAGAGGAAGTAA | *Platinum*™ *Direct PCR Universal Master Mix*-Kit | Gardes and Bruns (1993) |
|  | LR3 | CCGTGTTTCAAGACGGG |  | Friedl and Rokitta (1997) |
| LSU | LR7 | TACTACCACCAAGATCT | *Platinum*™ *Direct PCR Universal Master Mix*-Kit | Vigalys and Hester (1990) |
|  | LR0R | ACCCGCTGAACTTAAGC |  | Rehner and Samuels (1994) |
| RPB1 | RPB1-VJAFasc | ADTGYCCYGGYCATTTYGGT | (Vilgalys und Hester 1990) | Hofstetter et al. (2007) |
|  | RPB1-VH6R | ATGACCCATCATRGAYTCCTTRTG |  |  |
| RPB2 | fRPB2-5F | GAYGAYMGWGATCAYTTYGG | (Spjut et al. 2020) | Liu et al. (1999) |
|  | fRPB2-7CR | CCCATRGCTTGYTTRCCCAT |  |  |
| ***Roccellinastrum spongoideum*** | | | | |
| mtSSU | mrSSU1 | AGCAGTGAGGAATATTGGTC | *Platinum*™ *Direct PCR Universal Master Mix*-Kit | Zoller et al. (1999) |
|  | mrSSU3R | ATGTGGCACGTCTATAGCCC |  |  |
| ITS | ITS1f | CTTGGTCATTTAGAGGAAGTAA | *Platinum*™ *Direct PCR Universal Master Mix*-Kit | Gardes and Bruns (1993) |
|  | LR3 | CCGTGTTTCAAGACGGG |  | Friedl and Rokitta (1997) |
| RPB1 | RPB1-VJAFasc | ADTGYCCYGGYCATTTYGGT | (Vilgalys und Hester 1990) | Hofstetter et al. (2007) |
|  | RPB1-VH6R | ATGACCCATCATRGAYTCCTTRTG |  |  |
| RPB2 | fRPB2-5F | GAYGAYMGWGATCAYTTYGG | (Spjut et al. 2020) | Liu et al. (1999) |
|  | fRPB2-7CR | CCCATRGCTTGYTTRCCCAT |  |  |
| ***Photobionts*** | | | | |
| 18S | Al1500af | GCGCGCTACACTGATGC | *Platinum*™ *Direct PCR Universal Master Mix*-Kit | Helms et al. (2001) |
|  | LR3 | CCGTGTTTCAAGACGGG |  | Friedl and Rokitta (1997) |

**References:**

Friedl T, Rokitta C (1997) Species Relationships in the Lichen Alga *Trebouxia* (Chlorophyta, Trebouxiophyceae): Molecular Phylogenetic Analyses of Nuclear-Encoded Large Subunit rRNA Gene Sequences. Symbiosis, S. 125–148.

Gardes M, Bruns TD (1993) ITS primers with enhanced specificity for basidiomycetes-application to the identification of mycorrhizae and rusts. Molecular ecology 2 (2), S. 113–118. DOI: 10.1111/j.1365-294X.1993.tb00005.x.

Helms G, Friedl T, Rambold G, Mayrhofer H (2001) Identification of Photobionts from the lichen family Physciaceae using algal-specific ITS rDNA sequencing. The Lichenologist 33 (1), S. 73–86. DOI: 10.1006/lich.2000.0298.

Hofstetter V, Miadlikowska J, Kauff F, Lutzoni F (2007) Phylogenetic comparison of protein-coding versus ribosomal RNA-coding sequence data: a case study of the Lecanoromycetes (Ascomycota). Molecular phylogenetics and evolution 44 (1), S. 412–426. DOI: 10.1016/j.ympev.2006.10.016.

Liu YJ, Whelen S, Hall BD (1999) Phylogenetic relationships among ascomycetes: evidence from an RNA polymerse II subunit. Molecular biology and evolution 16 (12), S. 1799–1808. DOI: 10.1093/oxfordjournals.molbev.a026092.

Rehner SA, Samuels GJ (1994) Taxonomy and phylogeny of *Gliocladium* analysed from nuclear large subunit ribosomal DNA sequences. Mycological research 98 (6), S. 625–634. DOI: 10.1016/S0953-7562(09)80409-7.

Spjut R, Simon A, Guissard M, Magain N, Sérusiaux E (2020) The fruticose genera in the Ramalinaceae (Ascomycota, Lecanoromycetes): their diversity and evolutionary history. MycoKeys 73, S. 1–68. DOI: 10.3897/mycokeys.73.47287.

Vilgalys R, Hester M (1990) Rapid genetic identification and mapping of enzymatically amplified ribosomal DNA from several *Cryptococcus* species. Journal of bacteriology 172 (8), S. 4238–4246. DOI: 10.1128/jb.172.8.4238-4246.1990

White TJ, Bruns TD, Lee SB, Taylor JW (1990) Amplification and direct sequencing of fungal ribosomal RNA genes for phylogenetics. PCR Protocols: a Guide to Methods and Applications (M. A. Innis, D. H. Gelfand, J. J. Sninsky & T. J. White, eds): 315–322. San Diego: Academic Press.

Zoller S, Scheidegger C, Sperisen C (1999) PCR Primers for the Amplification of Mitochondrial Small Subunit Ribosomal DNA of Lichen-forming Ascomycetes. The Lichenologist 31 (5), S. 511–516. DOI: 10.1006/lich.1999.0220.
