# Supplementary material for: ﻿Roccellinastrum, Cenozosia and Heterodermia: Ecology and phylogeny of fog lichens and their photobionts from the coastal Atacama Desert
Source: MycoKeys. 2023 Aug 1;98:317–48. doi: 10.3897/mycokeys.98.107764 (PMC10410537; doi:10.3897/mycokeys.98.107764)
Supplement: Supplementary material 2 — Accession numbers used for the concatenated alignment of Heterodermia [file mycokeys-98-317-s002.docx]

| **Spezies** | **Collection** | **ITS1-ITS4** | **LR5-LRO5** | **mrSSU3r-mrSSU1** |
| --- | --- | --- | --- | --- |
| **Heterodermia adunca** | **LW01** | **OR042204** | **OR044081** | **OR042194** |
| **Heterodermia adunca** | **LW02** | **OR042205** | **OR044082** | **OR042195** |
| **Heterodermia follmannii** | **LW03** | **OR042206** | **OR044083** | **OR042196** |
| **Heterodermia follmannii** | **LW04** | **OR042207** | **OR044084** | **OR042197** |
| Heterodermia albicans | 49538 | MG257791 | MG257791 |  |
| Heterodermia speciosa | MP34 | KX512927 | KX512868 |  |
| Heterodermia speciosa | O-L-176824 | MK811755 |  | KX512975 |
| Heterodermia speciosa | Wetmore 88030 (UPS) | JX000105 | JX000089 |  |
| Heterodermia speciosa | L58942 (S) |  |  | JX000125 |
| Klauskalbia obscurata | BCN-Lich 15527 | GU247151 |  | GU247185 |
| Klauskalbia obscurata | BCN-Lich 16794 | GU247152 |  | GU247186 |
| Klauskalbia obscurata | Hur 040623 | EU045436 |  | KM397361 |
| Leucodermia boryi | D342 HL2 | KU862951 |  |  |
| Leucodermia boryi | Hur 040340 | EU045422 |  | KM397357 |
| Leucodermia boryi | Hur 040754 | EU045423 |  | KM397356 |
| Leucodermia boryi | S2 11 HL3 | KU862952 |  |  |
| Leucodermia erinacea | 2A/2B | EF582746 |  | EF582789 |
| Leucodermia leucomelaena | MAF 7638 | AY449725 |  | AY464072 |
| Leucodermia leucomelaena | 44717 | MK092093 | MK092093 |  |
| Leucodermia subascendens | Hur 040619 | EU045441 |  | KM397362 |
| Leucodermia subascendens | Hur 040658 | EU045427 |  |  |
| Leucodermia subascendens | Hur 040847 | EU045442 |  | KM397363 |
| Leucodermia subascendens | KoLRI 30895 | OK138537 |  |  |
| Leucodermia vulgaris | AFTOL-ID 320 | HQ650704 | DQ883798 | DQ912288 |
| Leucodermia vulgaris | HV | KX512928 |  | KX512989 |
| Polyblastidium casarettianum | 15441a | DQ337305 |  |  |
| Polyblastidium hypoleucum | Hur 060232 | EU045428 |  | KM397358 |
| Polyblastidium hypoleucum | Hara Kojiro: 0034 | LC533098 |  |  |
| Polyblastidium japonicum |  | AY498687 |  |  |
| Polyblastidium japonicum | Hur 060291 | EU045432 |  | KM397359 |
| Polyblastidium japonicum | Hur 061140 | KM397355 |  |  |
| Polyblastidium microphyllum | Hur 060173 | EU045433 |  | KM397360 |
| Polyblastidium microphyllum | Hur 060494 | EU045434 |  |  |
| Polyblastidium propaguliferum | Hur 041134 | EU045437 |  |  |
| Polyblastidium sp. | 16606a | DQ337327 |  |  |
| Xanthoria sp. | Gaya 31 | JQ301692 | JQ301591 | JQ301532 |
