# Supplementary material for: ﻿Roccellinastrum, Cenozosia and Heterodermia: Ecology and phylogeny of fog lichens and their photobionts from the coastal Atacama Desert
Source: MycoKeys. 2023 Aug 1;98:317–48. doi: 10.3897/mycokeys.98.107764 (PMC10410537; doi:10.3897/mycokeys.98.107764)
Supplement: Supplementary material 3 — Accession numbers used for the concatenated alignment of Cenozosia [file mycokeys-98-317-s003.docx]

| **Collection** | **ITS** | **rpb1** | **rpb2** |
| --- | --- | --- | --- |
| **LW09** | **OR042202** | **OR242358** | **OR242364** |
| **LW10** | **OR042203** | **OR242359** | **OR242365** |
| **LW07** | **OR042200** | **OR242356** | **OR242362** |
| **LW08** | **OR042201** | **OR242357** | **OR242363** |
| F. Lutzoni | DQ782850 | DQ782830 | DQ782872 |
| 3948 | MG926002 | MG926196 | MG926269 |
| 3157 | AF282093 |  | DQ992423 |
| 23757 | AF282070 |  | AM292758 |
| LW07 |  |  |  |
| LW08 |  |  |  |
| LW09 |  |  |  |
| LW10 |  |  |  |
| 3115 | MG925966 | MG926166 | KF662436 |
| 137 |  |  |  |
| 196 | MN811239 |  |  |
| T590 | MN811387 |  |  |
| 870 | MN811252 |  |  |
| 10207 | MG925969 | MG926169 | MG926249 |
| 480491 | AY584651 | DQ912379 | DQ912401 |
| 26038 | MG925985 | AY756419 | MG926258 |
| 5006 | MN811340 |  |  |
| 6056 | MN811372 |  |  |
| 4748 | MN811297 | MN757092 | MN757286 |
| 4878 | MN811336 | MN757128 | MN757317 |
| 4880 | MN811338 | MN757130 |  |
| 4783 | MN811307 | MN757100 | MN757293 |
| 4879 | MN811337 | MN757129 | MN757318 |
| 5101 | MN811354 | MN757145 | MN757331 |
| 6250 | MN811377 | MN757165 |  |
| 4688 | MN811289 | MN757086 |  |
| 4840 | MN811319 | MN757112 |  |
| 4677 | MN811286 | MN757084 | MN757279 |
| 4883 | MN811413 | MN757197 |  |
| 5120 | MN811361 | MN757152 | MN757336 |
| 4855 | MN811411 | MN757195 |  |
| 604 | GU827335 | MN757033 | MN757243 |
| 4877 | MN811335 | MN757127 | MN757316 |
| 5054 | MN811431 | MN757213 | MN757379 |
| 4702 | MN811292 | MN757088 | MN757283 |
| 4751 | MN811298 | MN757093 | MN757287 |
| 4768 | MN811301 | MN757096 | MN757289 |
| 5081 | MN811419 | MN757202 | MN757369 |
| 4871 | MN811333 | MN757125 | MN757314 |
| 421 |  |  |  |
| 4858 | MN811327 | MN757120 | MN757309 |
| 4886 | MN811414 | MN757198 | MN757367 |
| 5080 | MN811418 | MN757201 | MN757368 |
| 4866 | MN811331 | MN757123 | MN757312 |
| 4767 | MN811300 | MN757095 | MN757288 |
| 4814 | MN811315 | MN757108 | MN757298 |
| 4863 | MN811330 |  |  |
| 4731b | MN811425 | MN757208 | MN757375 |
| 4752 | MN811299 | MN757094 |  |
| 4788 | MN811310 | MN757103 |  |
| 5033 | MN811341 | MN757132 | MN757320 |
| 5130 | MN811366 | MN757157 | MN757341 |
| 4774 | MN811303 | MN757097 | MN757290 |
| 4777 | MN811304 | MN757098 | MN757291 |
| 4719 | MN811401 | MN757187 | MN757358 |
| 10275 | MG925994 | MG926189 | MG926189 |
| 515 | MG925991 | MG926186 | MG926263 |
| RE0810/2 | MG925992 | MG925992 |  |
| SK1-108 | MG925967 | MG926167 | MG926247 |
| CG659 | HQ650618 | DQ986830 | DQ992446 |
| 47560 | HQ650620 | DQ986831 | DQ992448 |
| 303 | GU827303 | MN757001 |  |
| 351 | GU827311 | MN757009 |  |
| 430 | GU827318 | MN757016 | MN757231 |
| 1509 | MN811248 | MN757055 | MN757260 |
| 3136 | MN811280 | MN757083 |  |
| 526 | GU827326 | MN757027 | MN757238 |
| 544 | GU827329 | MN757047 | MN757255 |
| 174 | MN811238 | MN757038 | MN757248 |
| 503 | MN811242 | MN757041 |  |
| 176 | GU827299 | MN756997 | MN757221 |
| 241 | MN811240 | MN757039 | MN757249 |
| 508 | MN811243 | MN757042 | MN757250 |
| 162 | GU827297 | MN756995 | MN757219 |
| 373 | GU827312 | MN757010 | MN757225 |
| 1551 | MN811269 | MN757067 | MN757270 |
| 84 | GU827295 | MN756993 |  |
| 1519 | MN811263 | MN757061 | MN757266 |
| 2111 | MN811276 | MN757074 | MN757274 |
| 541 | MN811247 | MN757046 | MN757254 |
| AFTOL 86 | HQ650720 | DQ973059 | DQ973072 |
| 1526 | MN811267 | MN757065 |  |
| 2113 | MN811278 | MN757076 | MN757276 |
| 4681 | MN811287 |  |  |
| 4772 | MN811302 |  |  |
| Nash 40171 |  | MG926127 | MG926224 |
| 3 | GU827289 | MN756987 |  |
| 445 | GU827320 | MN757018 | MN757233 |
| 343 | GU827309 | MN757007 |  |
| 65 | GU827291 | MN756989 | MN757215 |
| 326 | GU827305 | MN757003 |  |
| 374 | GU827313 | MN757011 | MN757226 |
| 132 | GU827296 | MN756994 | MN757218 |
| 1555 | MN811272 | MN757070 |  |
| 375 | GU827314 | MN757012 | MN757227 |
| 520 | MN811244 | MN757043 | MN757251 |
| 528 | MN811245 | MN757044 | MN757252 |
| 532 | MN811246 | MN757045 | MN757253 |
| 6012 | MN811370 | MN757161 | MN757344 |
| 6013 | MN811371 |  | MN757383 |
| 656 | GU827332 | MN757030 | MN757241 |
| 867 | MN811251 | MN757050 |  |
| 349 | GU827310 | MN757008 |  |
| 1552 | MN811270 | MN757068 |  |
| 448 | GU827321 | MN757019 |  |
| 513 | GU827328 | MN757026 | MN757237 |
| 1503 | MN811256 | MN757054 | MN757259 |
| 1513 | MN811260 | MN757058 | MN757263 |
| 72 | GU827290 | MN756988 |  |
| 2846 | MN811283 | MN757081 |  |
| 456 | GU827322 | MN757020 |  |
| 242 | GU827301 | MN756999 | MN757222 |
| 1553 | MN811427 |  |  |
| 428 | GU827317 | MN757015 | MN757230 |
| 340 | GU827308 | MN757006 | MN757224 |
| 501 | GU827324 | MN757022 | MN757235 |
| 2668 | MN811281 | MN757079 |  |
| 2964 |  |  |  |
| 462 | GU827323 | MN757021 | MN757234 |
| 175 | GU827298 | MN756996 | MN757220 |
| 509 | GU827327 | MN757025 |  |
| 1169 | MN811253 | MN757051 | MN757258 |
| 1514 | MN811261 | MN757059 | MN757264 |
| 1561 | MN811274 | MN757072 | MN757272 |
| 2112 | MN811277 | MN757075 | MN757275 |
| 422 | GU827315 | MN757013 | MN757228 |
| 431 | GU827319 | MN757017 | MN757232 |
| 459 | MN811241 | MN757040 |  |
| 529 | GU827331 | MN757029 | MN757240 |
| 666 | MN811250 | MN757049 | MN757257 |
| 426 | GU827316 | MN757014 | MN757229 |
| 82 | GU827294 | MN756992 | MN757217 |
| 1558 | MN811257 | MN757071 | MN757271 |
| 2114 | MN811273 | MN757077 | MN757277 |
| 2115 | MN811279 | MN757078 | MN757278 |
| 504 | MN811285 | MN757024 | MN757236 |
| 2669 | MN811282 | MN757080 |  |
| 1554 | MN811271 | MN757069 |  |
| 527 | GU827330 | MN757028 | MN757239 |
| 546 | MN811249 | MN757048 | MN757256 |
| 1525 | MN811266 | MN757064 | MN757269 |
| 1702 | MN811275 | MN757073 | MN757273 |
| 1550 | MN811268 | MN757066 |  |
| 1460 | MN811254 | MN757052 |  |
| 1476 | MN811255 | MN757053 |  |
| 338 | GU827307 | MN757005 |  |
| 502 | GU827325 | MN757023 |  |
| 57 | MN811237 | MN757037 | MN757247 |
| 179 | GU827300 | MN756998 |  |
| 249 | GU827302 | MN757000 |  |
| 331 | GU827306 | MN757004 | MN757223 |
| 68 | GU827292 | MN756990 | MN757216 |
| 1510 | MN811258 | MN757056 | MN757261 |
| 1511 | MN811259 | MN757057 | MN757262 |
| 1516 | MN811262 | MN757060 | MN757265 |
| 1520 | MN811264 | MN757062 | MN757267 |
| 1522 | MN811265 | MN757063 | MN757268 |
| 73 | GU827293 | MN756991 |  |
| 306 | GU827304 | MN757002 |  |
| RE08/2 |  | MG926184 | MG926261 |
| 5688 | HQ650608 | DQ986835 | DQ992452 |
| 97-407 | AF282120 |  | DQ973073 |
| 5116 | MN811360 | MN757151 |  |
| 4841 | MN811320 | MN757113 | MN757303 |
| 777 | HQ650621 | DQ986832 | DQ992436 |
| 5050 | MN811345 | MN757136 | MN757324 |
| 4741 | MN811295 | MN757090 | MN757285 |
| 128 | GU827333 | MN757031 |  |
| 4828 | MN811430 | MN757212 | MN757378 |
| 5073 | MN811417 | MN757200 |  |
| 5134 | MN811367 | MN757158 | MN757342 |
| 5114 | MN811359 | MN757150 | MN757335 |
| 5107 | MN811420 | MN757203 | MN757370 |
| 4672B | MN811383 | MN757171 | MN757345 |
| 4714 | MN811293 | MN757089 | MN757284 |
| 5087 | MN811349 | MN757140 | MN757328 |
| 5104 | MN811355 | MN757146 | MN757332 |
| 5109 | MN811357 | MN757148 | MN757333 |
| 5056 | MN811346 | MN757137 | MN757325 |
| 4744 | MN811296 | MN757091 |  |
| 4818 | MN811316 | MN757109 | MN757299 |

The original dataset is from:

Spjut, R., Simon, A., Guissard, M., Magain, N., & Sérusiaux, E. (2020). The fruticose genera in the Ramalinaceae (Ascomycota, Lecanoromycetes): their diversity and evolutionary history [Erratum: October 2020, Vol. 74, pp. 109-110].
