# Supplementary material for: ﻿Roccellinastrum, Cenozosia and Heterodermia: Ecology and phylogeny of fog lichens and their photobionts from the coastal Atacama Desert
Source: MycoKeys. 2023 Aug 1;98:317–48. doi: 10.3897/mycokeys.98.107764 (PMC10410537; doi:10.3897/mycokeys.98.107764)
Supplement: Supplementary material 4 — Spot tests and TLC [file mycokeys-98-317-s004.docx]

**Table 1: Results of TLC and spot test of *Roccellinastrum spongoideum*; +: is contained, ±: traces may be contained.**

|  | **TLC** | | | | | | **Spot Test** | | | | |
| --- | --- | --- | --- | --- | --- | --- | --- | --- | --- | --- | --- |
|  | Atranorin | Protocetric acid | Squmatic acid | Norstictic acid | Connorstictic acid | unknown | UV | C | K | P | K  C |
| ***R. spongoideum*** | + | + |  |  |  | + (2) |  |  |  | +  (yellow / orange) | +  (orange) |
| *R. candidum* | + | + |  |  |  | ± |  |  |  |  |  |
| *R. epiphyllum* | + | + |  | + | + |  |  |  | + (yellow) | + (orange) |  |
| *R. neglectum* |  | + | + |  |  | + |  |  |  |  |  |

**Table 2: Results of TLC and spot tests of *Heterodermia follmannii* and *Heterodermia adunca*; +: is contained, ±: may contain traces.**

|  | **TLC** | | | | | | **Spot Test** | | | | |
| --- | --- | --- | --- | --- | --- | --- | --- | --- | --- | --- | --- |
|  | Atranorin | Zeorin | Salazinic acid | Norstictic acid | 16β-acetoxyhopane-6α,22- diol | u  n  k  n  o  w  n | UV | C | K | P | K  C |
| ***Heterodermia follmannii*** | + | + |  |  |  |  |  |  | + (yellow) |  |  |
| ***Heterodermia adunca*** | + | + |  |  |  |  |  |  | +  (slightly yellow) |  |  |
| *Klauskalbia obscurata* | + | + |  |  | + |  |  |  | + (yellow) | Cortex: + (yellow)  Medulla: + (violet) |  |
| *Polyblastidium hypoleucum* | + | + |  | ± |  |  |  |  | + (yellow) |  |  |
| *Polyblastidium japonicum* | + | + | ± | ± |  | +  (Terpenes) |  |  | + (yellow) | + (orange) |  |
| *Heterodermia speciosa* | + | + |  |  |  | + (Terpenes) |  |  | + (yellow) |  |  |
| *Leucodermia leucomelaena* | + | + | ± |  |  |  |  |  | + (yellow) | + (gelb) |  |

**Table 3: Results of TLC and spot tests of *Cenozosia cava and C. excorticata*; +: is contained, ±: may contain traces**.

|  | **TLC** | | | | | | | | | **Spot Test** | | | | |
| --- | --- | --- | --- | --- | --- | --- | --- | --- | --- | --- | --- | --- | --- | --- |
|  | Zeorin | Salazinic acid | Hydroxykaurane | Decarboxynorstenosporic acid | Divaricatic acid | Decarboxydivaricatic acid | Terpenes | Fatty acid | Un  k  nown | U  V | C | K | P | K  C |
| ***Cenozosia cava*** | ± |  |  | + |  | + |  | + | + (2) |  | + (red) |  |  | +(yellow) |
| ***Cenozosia excorticata*** | ± |  |  | + |  | + |  |  | + (1) |  | + (red) |  |  |  |
| *Ramalina canariensis* |  |  |  |  | + |  |  |  |  |  |  |  |  |  |
| *Ramalina bourgeana* |  | + |  |  |  |  | + |  |  |  |  |  |  |  |
| *Niebla homalea* |  |  |  |  | + |  |  |  |  |  |  |  |  |  |
| *Niebla flagelliforma* |  |  |  |  | + |  |  |  |  |  |  |  |  |  |
| *Niebla flabellata* |  | + |  |  |  |  |  |  |  |  |  |  |  |  |
| *Vermilacinia cerebra* |  | + | + |  |  |  | + | + |  |  |  |  |  |  |
| *Vermilacinia procera* | + | + | + |  |  |  | + |  |  |  |  |  |  |  |
